# Single-cell analysis reveals *Mycobacterium tuberculosis* ESX-1–mediated accumulation of permissive macrophages in infected mouse lungs

**DOI:** 10.1126/sciadv.adq8158

**Published:** 2025-01-15

**Authors:** Weihao Zheng, Michael Borja, Leah C. Dorman, Jonathan Liu, Andy Zhou, Amanda Seng, Ritwicq Arjyal, Sara Sunshine, Alina Nalyvayko, Angela Oliveira Pisco, Oren S. Rosenberg, Norma Neff, Beth Shoshana Zha

**Affiliations:** ^1^ Division of Experimental Medicine, Department of Medicine, University of California, San Francisco, CA, USA.; ^2^ Chan Zuckerberg Biohub, San Francisco, CA, USA.; ^3^ Department of Biochemistry and Biophysics, University of California, San Francisco, CA, USA.; ^4^ Division of Infectious Diseases, Department of Medicine, University of California, San Francisco, California, USA.; ^5^ Division of Pulmonary, Critical Care, Allergy and Sleep Medicine, Department of Medicine, University of California, San Francisco, CA, USA.

## Abstract

*Mycobacterium tuberculosis* (MTB) ESX-1, a type VII secretion system, is a key virulence determinant contributing to MTB’s survival within lung mononuclear phagocytes (MNPs), but its effect on MNP recruitment and differentiation remains unknown. Here, using multiple single-cell RNA sequencing techniques, we studied the role of ESX-1 in MNP heterogeneity and response in mice and murine bone marrow–derived macrophages (BMDM). We found that ESX-1 is required for MTB to recruit diverse MNP subsets with high MTB burden. Further, MTB induces a transcriptional signature of immune evasion in lung macrophages and BMDM in an ESX-1–dependent manner. Spatial transcriptomics revealed an up-regulation of permissive features within MTB lesions, where monocyte-derived macrophages concentrate near MTB-infected cells. Together, our findings suggest that MTB ESX-1 facilitates the recruitment and differentiation of MNPs, which MTB can infect and manipulate for survival. Our dataset across various models and methods could contribute to the broader understanding of recruited cell heterogeneity during MTB lung infection.

## Teaser

Single-cell profiling reveals that *M. tuberculosis* ESX-1 drives recruitment and manipulation of immunoevasive macrophages.

## INTRODUCTION

*Mycobacterium tuberculosis* (MTB) is the agent responsible for tuberculosis, one of the world’s deadliest infectious diseases (*1*). Central to the pathogenicity of MTB is its remarkable ability to survive and replicate within innate immune cells (*2*, *3*). In the early innate immune stage of lung infection, alveolar macrophages (AMs) are the major infected cell type for MTB (*2*, *3*). Further, infected AMs adopt an anti-inflammatory signature that is less conducive to bacterial elimination (*2*, *4*). In this environment, MTB can replicate and induce the transfer of AMs to the interstitial compartment (*3*). Subsequently, neutrophils and monocytes are recruited to the site of infection, and in parallel, MTB disseminates to newly recruited phagocytes (*5*–*7*). In particular, Ly6c^hi^ monocytes represent the primary recruited population (*8*) and differentiate into macrophages and dendritic cells (DC) (*8*–*10*), which become the major MTB reservoir after the development of T cell responses (*11*, *12*). During chronic infection, bacterial burden begins to plateau after activation and recruitment of T cells but often does not result in eradication (*13*, *14*). Paradoxically during this later stage, recent evidence has unveiled that distinct subsets of mononuclear phagocytes (MNPs) are more permissive to MTB than other innate cells such as AMs (*12*). However, little is known about the host mechanisms supporting MTB to persist in vivo even in the presence of adaptive immune responses.

Characterization of MTB-permissive MNPs after the development of T cell responses has largely relied on using a combination of flow cytometry and MTB expressing fluorescent proteins (*3*, *9*, *11*, *12*, *15*). However, flow cytometry’s limitations—such as the use of predefined antibodies, finite fluorophores, and variable gating strategies (*3*, *8*)—have constrained the resolution of subset analysis and mechanistic understanding of the survival of intracellular MTB. Furthermore, inherent challenges lie in reconciling cellular subsets between the MTB-infected environment, homeostasis, and other chronic inflammatory states due to cellular plasticity and heterogeneity. For instance, CD11b^+^CD11c^+^ cells, previously defined as monocyte-derived (moDerived) DC (*8*, *9*, *16*), are heterogeneous and include moDerived macrophages (*10*, *11*). Therefore, other unbiased methods, such as single-cell RNA sequencing (scRNA-seq), are needed to better understand the cell types and cellular responses of MTB-permissive cells during chronic infection.

One central virulence factor contributing to MTB pathogenicity is early secretory antigenic target secretion system–1 (ESX-1), a type VII secretion system shown to be involved in multiple pathways including phagosome escape (*17*) and inhibition of phagolysosome maturation (*18*), and has been suggested to be involved in the recruitment of innate immune cells (*19*, *20*). Components of the ESX-1 machinery are primarily encoded in the operon region of difference 1 (RD1), which is lacking in the historic vaccine strain, Bacillus Calmette-Guerin (BCG) (*21*). While attenuated, MTB lacking functional ESX-1 can still survive, replicate, and spread cell-to-cell in mice after aerosol infection (*22*) but induce less myeloid cell recruitment (*6*, *12*). We hypothesize that ESX-1 drives the recruitment of heterogeneous MNP characterized by an attenuated anti-MTB response, which are conducive to MTB replication and dissemination.

Here, using different methods of scRNA-seq, we aimed to elucidate the role of MTB ESX-1 in MNP recruitment and heterogeneity, identify permissive subsets of MNP, and understand the spatial interaction of recruited bystander MNP, infected MNP, and the bacteria. Using aerosol infection, we compared uninfected mice, mice infected with MTB H37Rv, and mice infected with an attenuated H37Rv lacking functional ESX-1 machinery. We found that ESX-1–deficient H37Rv does not recruit and induce activation of macrophages to the same extent as the wild-type strain, and ESX-1 is required for recruitment of specific macrophage subsets conducive to MTB survival. Both in vitro and in vivo models revealed that ESX-1 is important for inducing immune evasion signals permissive for MTB survival. Using spatial transcriptomics, we also found an up-regulation of key permissive signals in MTB lesions, where moDerived macrophages concentrate near MTB-infected cells.

## RESULTS

### MTB recruits heterogeneous subsets of MNPs

**Fig. 1.**
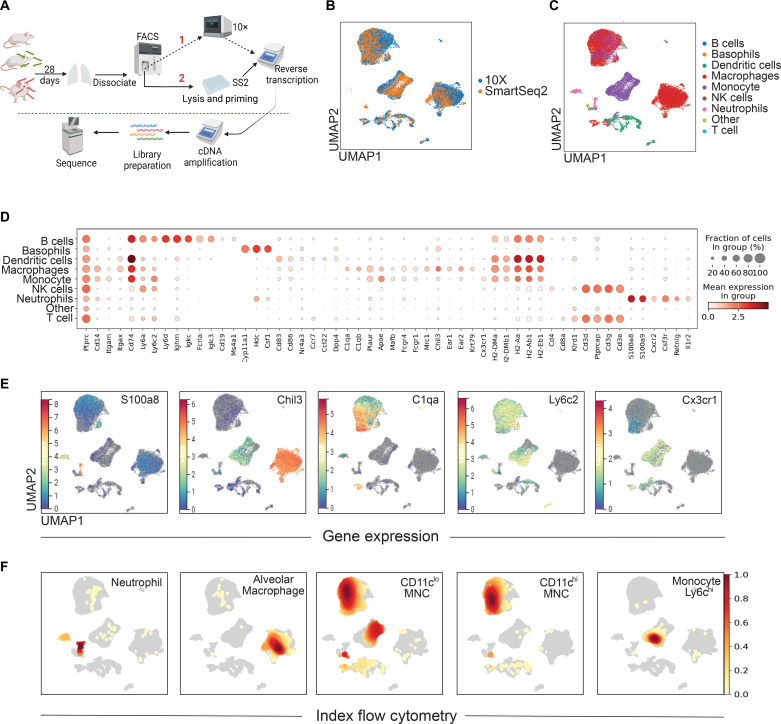
MTB induces recruitment of heterogeneous MNPs in the lungs of mice. (**A**) Mice were infected with MTB for 28 days, lung dissociated to single-cell suspension, and live cells flow-sorted to obtain myeloid cells differentiated by infection status based on bacterial fluorescence expression (fig. S1). Cells were either sorted directly into 384-well plates (path 1; SS2: SmartSeq2) or washed and counted for 10X chemistry (path 2). After reverse transcription and decontamination, samples were removed from the BSL3 for library preparation and sequencing. (**B**) Ingest was used to combine SmartSeq2 and 10X sequencing datasets into a single UMAP plot. (**C**) Cellular subsets were classified using the Immgen database within SinglR for unbiased annotation. (**D**) Dot plot shows transcriptional canonical markers where fraction of cells expressing the gene is denoted by size of dot, and the darker red denotes higher mean gene expression of the gene. (**E**) Gene expression of key canonical markers after log transformation and normalization visualized using CELLxGENE, where darker red signifies higher expression. (**F**) Density plots of subsets obtained through index sorting for SmartSeq2 using the MFI of surface markers were converted using boolenization and overlayed onto the transcriptional data. Five mice per condition as biological replicates, with each condition replicated three times technically, for lung cell sorting.

Previous work has demonstrated that following MTB infection, moDerived cells are recruited to the lungs and undergo differentiation over time (*8*, *10*). Recent work demonstrates that soon after the development of T cell responses, monocyte-derived lung cells (MNCs) are more permissive to MTB survival compared with AM (*11*, *12*). Despite best efforts at refined profiling, these cell subsets defined by flow cytometry remain heterogeneous. To investigate the differential properties of these and other MNP cells in early chronic infection (*23*), we used scRNA-seq. Mice were infected with H37Rv expressing mCherry and euthanized 28 days later to allow the development of the adaptive T cell response. After gating out natural killer (NK), T, and B cells, live cells were sorted by mCherry expression (i.e., positive indicative as infected or negative indicative as bystander; fig. S1A). Live cells were processed using the 10X genomic platform on live cells within a biological safety level 3 (BSL3) laboratory (Fig. 1A). Hierarchical Leiden clustering of cellular transcriptional libraries revealed multiple discrete cellular populations; however, heterogeneity of cell subsets was not delineated despite computational subclustering. We turned to SmartSeq2, a plate-based scRNA-seq platform in which single cells are directly sorted into a gentle lysis buffer (*24*). To capture cells of highest interest, single lung cells were sorted for CD11b and/or CD11c positivity into 384-well plates based on fluorescence expression (Fig. 1A and fig. S1B). As compared to 10X, SmartSeq2 demonstrated higher total counts with a 400-fold increase of total counts per cell, less ribosomal RNA capture, and more distinct delineation of unique moDerived cell subtypes (fig. S2). The higher counts in SmartSeq2 are partly due to its increased sequencing depth, the absence of deduplicating unique molecular identifiers in 10X data, and a higher capture rate that enhances per gene coverage in the SmartSeq2 preparation.

Given the higher number of cells captured, data obtained from SmartSeq2 were integrated with 10X for unbiased annotation using SinglR with mouse ImmGen database as reference (Fig. 1, B and C) (*25*). Nomenclature was confirmed by the expression of key canonical transcriptional markers (Fig. 1, D and E). In addition, index sorting during SmartSeq2 provided single-cell mean fluorescent intensity (MFI) analysis of antigen abundance. MFI of key surface markers used during gating was overlayed onto transcriptional cell clusters using Boolean computation (Fig. 1F, fig. S1D, and data S1). Clear identification of AMs (CD11c^+^SiglecF^+^, *Chil3*), neutrophils (Ly6g^+^, *S100a8* high), and monocytes (Ly6c^hi^, *Cx3cr1*^+^) could be delineated. Further, the majority of CD11c^hi^-MNC corresponded to the transcriptional annotation of macrophages, while CD11c^lo^-MNC contained both macrophages and monocyte-like cells. Together, index sorting and SmartSeq2 confirm the identification of heterogenous MNP at both mRNA and protein levels in early chronic MTB-infected mouse lungs.

### Lack of a functional ESX-1 prevents recruitment of diverse mDerived macrophages

**Fig. 2.**
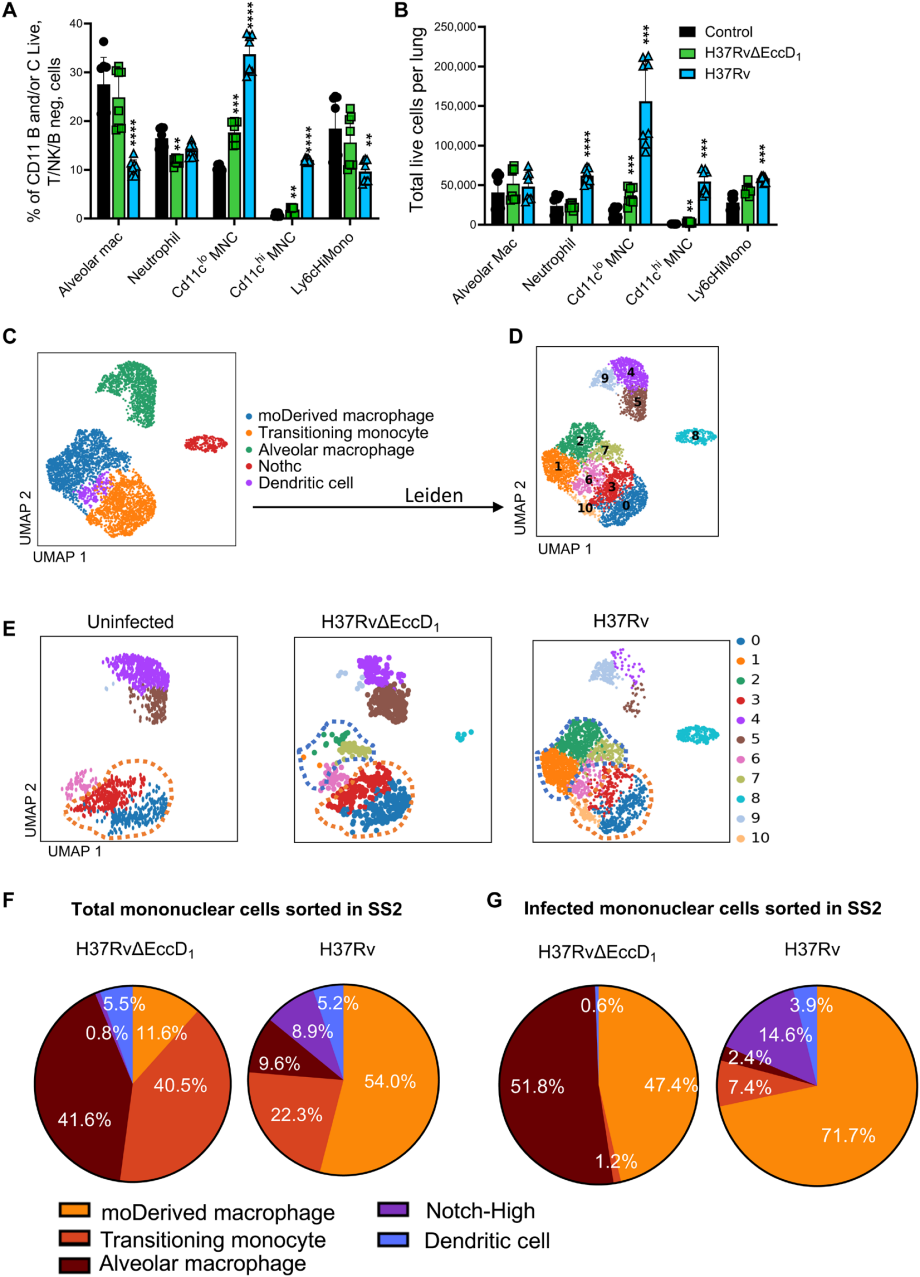
SmartSeq2 reveals ESX-1 recruitment of diverse subsets of moDerived macrophages. (**A** and **B**) C57BL/6 mice were infected for 28 days with H37Rv or H37RvΔEccD_1_, and lung MNPs were analyzed by flow cytometry compared to uninfected control mice. (**C**) SmartSeq2 analysis of CD11b- and/or CD11c-positive cells. (**D**) Cellular subsets were further clustered using Leiden algorithm. (**E**) Separation by infection status of the mouse that the cells were isolated, with moDerived macrophages denoted by blue hashtag and transitional cells by an orange. Percent cell type make-up was determined for both (**F**) total mononuclear cells sorted per infection condition and (**G**) of all infected mononuclear cells per condition. **P < 0.01, ***P < 0.001, and ****P < 0.0001 using *t* test with Welch correction and Holm-Sidak multiple comparisons. For (A) and (B), flow cytometry analysis was performed with three to six mice per group for biological replicates with a minimum of two technical replicates across four separate experiments capturing a minimum of 100,000 events per condition per sort.

ESX-1 plays an important role in myeloid cell recruitment and bacterial cell-to-cell transfer. However, mice can successfully be infected with MTB lacking functional ESX-1 (*6*, *12*, *22*). We next asked if functional depletion of ESX-1 would result in alteration of the observed heterogeneous recruitment of MNP, which may be a cause of alteration of the dynamics of bacterial cell-to-cell transfer (*6*, *12*). EccD_1_, a critical component of the ESX-1 complex formation (*26*), was deleted using phage-mediated allelic exchange (*27*). EccD_1_ was targeted rather than using the common H37RvΔRD1 strain to ensure that ESX-1 was the intended mutant rather than other potential virulence factors (fig. S3A) (*28*–*30*). Bacteria were confirmed to no longer secrete CFP-10 and ESAT-6, despite the expression of the proteins (fig. S3B). As expected, bacteria had a substantial decrease in virulence, as noted by a 200-fold lower CFU in the lungs of mice aerosol infected with H37RvΔRD1 compared to H37Rv (*P* < 0.01) (fig. S3C). Further, when used to infect mice, MTB lacking EccD_1_ resulted in fewer CD11c^hi^-MNC, CD11c^lo^-MNC, and neutrophils by flow cytometry (Fig. 2, A and B), comparable to findings when RD1 is deleted (*12*).

Given that cells infected with H37Rv lacking the operon RD1 during aerosol infection are rarely sorted (*6*), we used SmartSeq2 to capture rare events and thoroughly analyze recruited cell subsets. Mice were infected with MTB expressing zsGreen rather than mCherry to improve delineation of infected and bystander cells as H37RvΔEccD_1_-infected cells are rare. In addition, while lung perfusion substantially reduces the presence of intravascular cells, an intravenous injection of anti-CD45 was added as an additional measure to ensure that cells did not originate from the intravascular space (fig. S1C). Live cells were sorted to include all CD11b- and/or CD11c-positive cells not including NK/T/B cells, and to confirm aligned cellular identification, transcriptomic data were overlayed using Ingest (Scanpy) with the main atlas (fig. S3C). In line with flow cytometric results, the proportion of cell types present depended on infection status, with the most of the cells identified as AMs in the uninfected mouse, recruited MNPs from H37Rv-infected mice, and a mixture in the proportion of AMs and recruited cells in H37RvΔEccD_1_-infected mice.

Monocytes, macrophages, and DCs from both SmartSeq2 experiments were subset from the larger atlas to standardize the method, sequencing depth, and further factors that cannot be compensated computationally (fig. S3E). Cells were reclustered and noted to have similar distributions as the combined cell atlas. With this method, five clusters were identified: moDerived macrophages, transitioning monocytes, AMs, moDerived cells with elevated Notch signaling and underrepresentation of canonical markers (“Notch-High”), and a smaller DC population. Nomenclature was again confirmed by transcriptional canonical markers and previously defined surface markers (Fig. 2C and fig. S4, A and B) (*12*). For instance, the denotation transitioning monocyte was given due to a high expression of monocyte markers (e.g., *Cx3cr1*, *Sell*, *Spn*, *Plac8*, and *Nr4a1*) with a further signature of *C1q*, *Cd68*, *Csf1r*, and *Lyz2* (fig. S4A). Given experimental methods to exclude intravascular monocytes combined with transcriptional similarities, those with high Ly6c surface expression are also expected to be recently recruited or patrolling monocytes (*31*). Conversely, moDerived macrophages demonstrated significantly lower expression of monocyte markers while retaining *Ly6c2*, *Cd14*, major histocompatibility complex II (MHC II), and *C1q* markers.

Subsetting was further accomplished through CELLxGENE and Leiden clustering (Fig. 2D) (*32*, *33*), revealing three subsets each of moDerived macrophages (clusters 1, 2, and 7), transitioning monocytes (clusters 0, 3, and 10), and AMs (clusters 4, 5, and 9), each with differential infection states (Fig. 2, E to G). As hypothesized, mice infected with H37RvΔEccD_1_ had significantly fewer total infected cells that could be sorted compared to H37Rv (fig. S1C). The major infected subset in H37Rv-infected mice was moDerived macrophages, consistent with our previous study (*12*). In contrast, 51.8% of the infected cells in H37RvΔEccD_1_-infected cells were AM, and only 47.4% were moDerived macrophages (Fig. 2G). Notably, there were significantly fewer moDerived macrophages in the lungs of H37RvΔEccD_1_-infected mice (table S1). Similarly, there was a reverse in the proportion of moDerived macrophages to transitioning monocytes in H37RvΔEccD_1_-infected mice (11.6% versus 40.5% of all cells respectively, *P* < 0.0001) as compared to H37Rv-infected (54% versus 22.3% of all cells respectively, *P* < 0.0001). Therefore, the lack of ESX-1 resulted in a lack of moDerived macrophages despite the recruitment of monocytes.

### H37Rv with functional ESX-1 induces recruitment of permissive macrophages with a signature of immune evasion

**Fig. 3.**
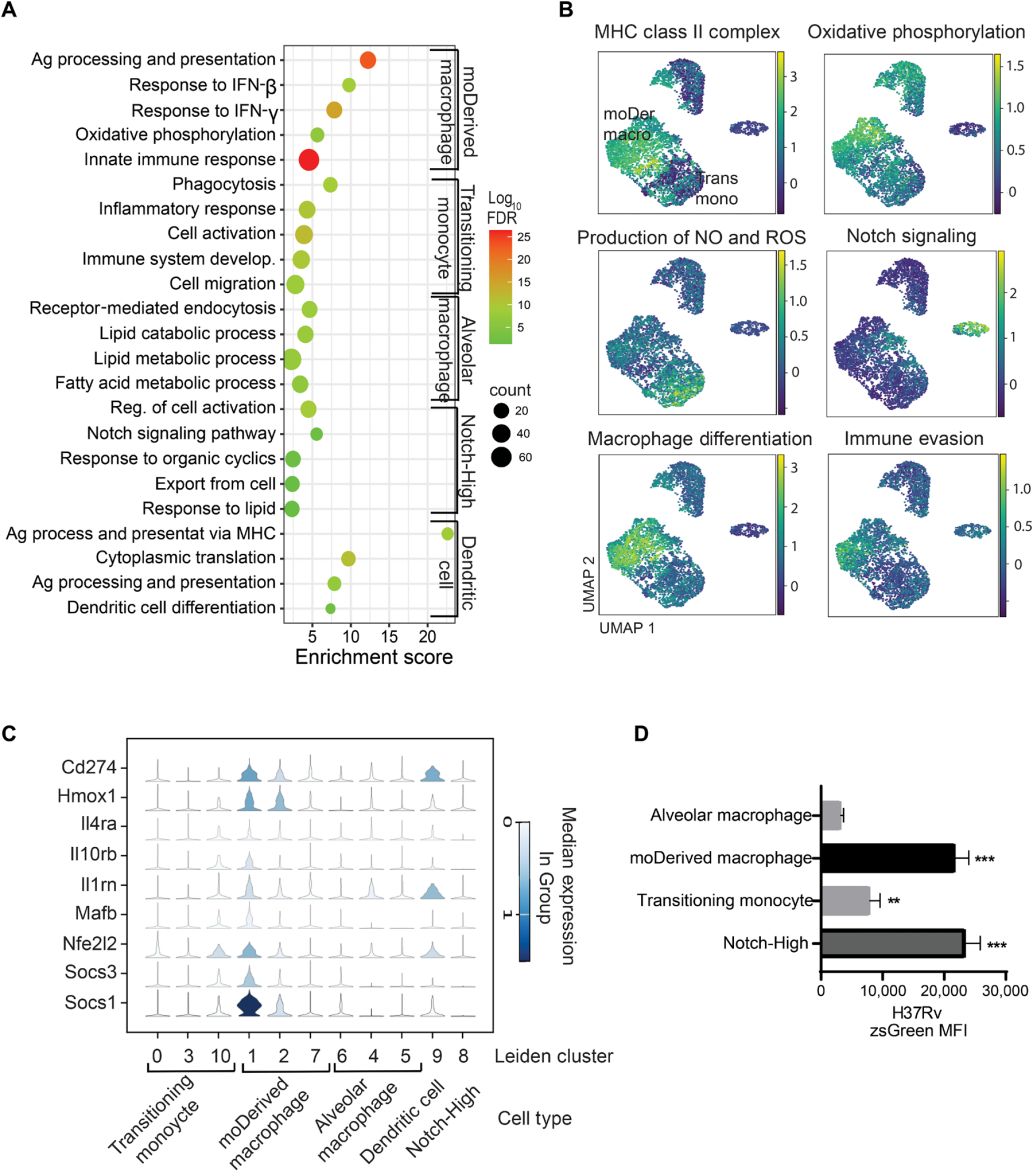
Recruited MNPs during lung MTB infection include a subset of cells with an anti-inflammatory signature. (**A**) Differentially expressed marker genes identified in each cell cluster were used for Gene Ontology analysis with ShinyGOv0.77. In the dot plot, color indicates the negative log-transformed FDR, while dot size corresponds to the number of genes enriched in each pathway. (**B**) Gene sets were constructed to score the likelihood of a particular cell with transcriptional enrichment of the denoted pathway using the average expression of associated genes (Methods). Lighter color denotes higher expression of gene set and thus predicted attribute. moDer Macro, moDerived macrophage (Leiden clusters 1, 2, and 7); Trans Mono, transitioning monocyte (Leiden clusters 0, 3, and 10). (**C**) Violin plot of differentially expressed genes from the “anti-inflammatory” pathways (table S2) as delineated by Leiden cluster. (**D**) ZsGreen MFI of infected MNP subsets from H37Rv/ZsGreen-infected mouse lungs (28 days post infection). Ag, antigen; NO, nitric oxide; ROS, reactive oxygen species.

We next turned to understanding the transcriptional differences of MNPs recruited during MTB infection. Differentially expressed genes were determined using MAST to reduce bias by adjusting the gene expression rate (*34*), and Gene Ontology and pathway enrichment were conducted only in those meeting a false discovery rate (FDR) of <0.05 (data S2). We found enrichment of oxidative phosphorylation and response to interferon (IFN) in moDerived macrophages compared with transitioning monocytes. Conversely, transitioning monocytes were enriched for pathways important in cellular activation and cell migration (Fig. 3A, fig. S4C, and data S3).

Diverse transcriptional responses between subclusters compared to all cells were noted repeatedly through analysis. To better visualize this heterogeneity, we applied gene enrichment scores to delineate phenotypic predictions. Scores were developed using genes from our experimental model in conjunction with previously defined and associated signatures (Fig. 3B, table S2, and Methods) (*35*). Here, AMs showed a diversity of transcriptional programs, with cluster 9 showing an up-regulation of antigen presentation compared to clusters 4 and 5, which had an up-regulation of endocytosis (Fig. 3B and fig. S4D). Transitioning monocytes in clusters 0 and 3 showed differentiation between a phagocytic gene program in cluster 0 compared to cluster 3. The Notch-High cluster separated from the two main clusters of moDerived cells markedly denoted by a higher ratio of H37Rv-infected cells (63% as compared to 50% in moDerived macrophages, *P* < 0.05; Fig. 2, E and F). Few transcriptional markers delineating the origin of cell type remained, and Notch signaling was repeatedly enriched in multiple analyses, with highly expressed genes such as *Notch2*, *Cdkn1b*, *Jag1*, *Adam10*, and *Dtx3l* (Fig. 3B and data S3).

Recruited moDerived macrophages (clusters 1, 2, and 7) demonstrated an up-regulation of MHC II complex that was enriched in cluster 1 and oxidative phosphorylation in cluster 7 (Fig. 3B). IFN-β signaling was enriched in recruited cells especially moDerived macrophages (fig. S4E), which is known to hamper MTB control in vivo (*36*). Because other immunosuppressive proteins, in addition to IFN-β, could suppress anti-mycobacterial activities in MNPs, we built an immune evasion signature in addition to the IFN-β signature (table S2) (*11*). When compared to AM and transitioning monocytes, there was a signal concentrated in moDerived macrophages cluster 1 from mice infected with H37Rv. Cluster analysis revealed that these genes are involved in an oxidative response and negative regulation of T cell activation and IFN-γ signaling (fig. S4F). In addition, the up-regulation of key immunoevasion-related genes, including *Hmox1* (*37*), *Il10* (*38*), *Il1rn* (Il1 receptor antagonist) (*36*, *39*), and *Nfe2l2* (Nrf2) (*2*), coupled with a lower score of “production of nitric oxide (NO) and reactive oxygen species (ROS),” suggests an impaired anti-MTB response and that recruited moDerived macrophages may be more conducive to MTB replication (Fig. 3C). Infected moDerived macrophages harbor more bacterial burden (indicated by MTB fluorescence) than infected AM (Fig. 3D), consistent with previous findings that moDerived cells contain more live bacteria (*12*). Collectively, these findings suggest that virulent MTB recruits permissive macrophages with a signature of immune evasion despite the development of adaptive immune responses.

### ESX-1 is vital for the transcriptional induction of both recruitment and maturation of macrophages of infected lungs

**Fig. 4.**
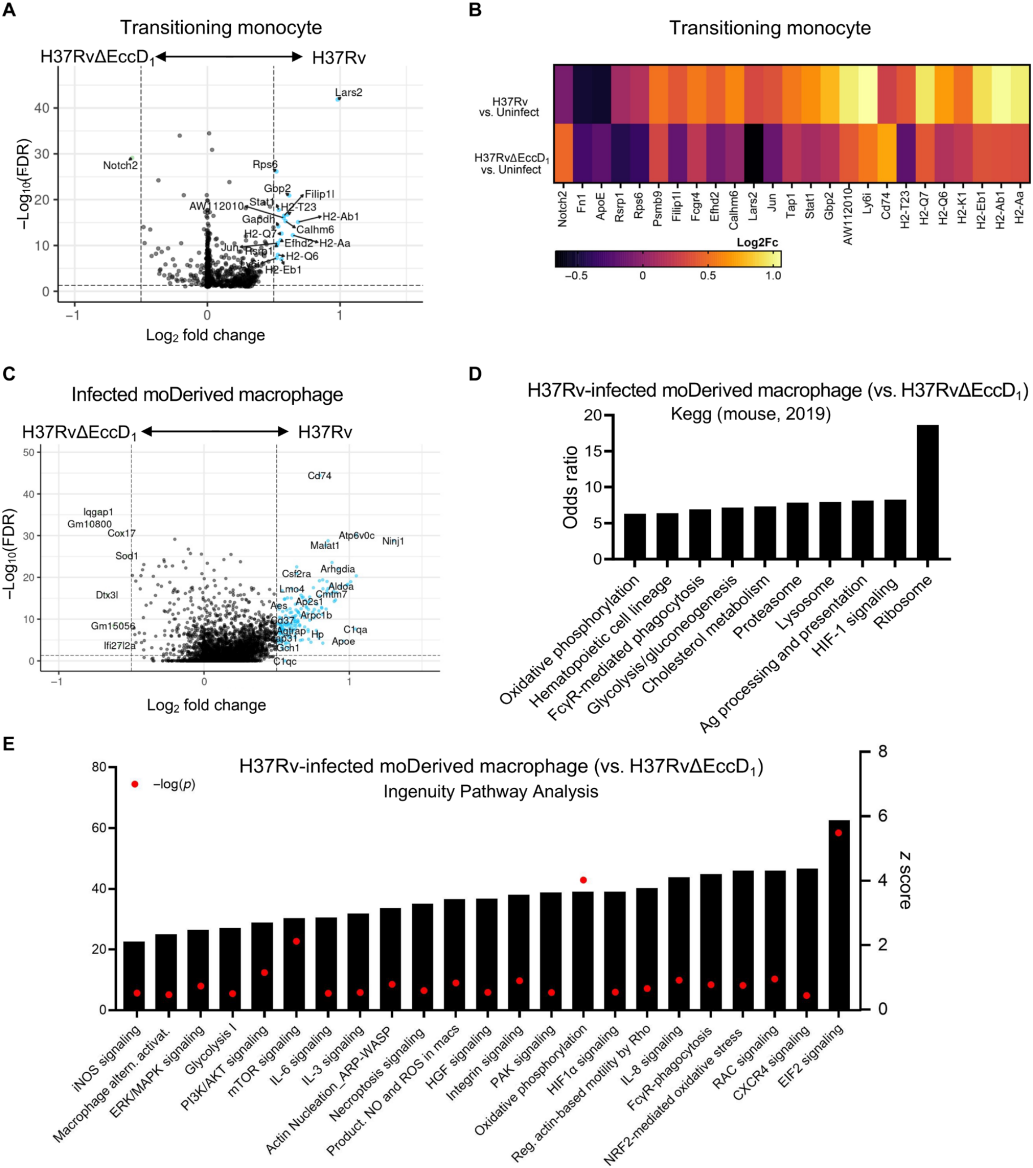
ESX-1 promotes macrophage activation and immune evasion response. (**A**) Volcano plot of transitioning monocytes from mice infected with H37Rv and H37RvΔEccD_1_ analyzed for differential gene expression differences using MAST. (**B**) Log_2_ fold change differences of differentially expressed genes between H37Rv versus H37RvΔEccD_1_ or each condition to cells present in the uninfected control mouse. (**C**) Volcano plot of differentially expressed genes in mature moDerived macrophages infected with H37Rv as compared to H37RvΔEccD_1_. (**D**) Odds ratio for pathway enrichment using KEGG (mouse 2019), with the top 10 statistically significant pathways shown, removing redundancy. (**E**) QIAGEN IPA was also queried using differentially expressed genes determined from MAST analysis with an FDR < 0.1. Pathways shown are up-regulated, with a *z* score on the right *x* axis. The −log(FDR) is shown by red dots with values depicted on the left *x* axis. Fc, fold change.

Given that the lack of ESX-1 activity resulted in significantly fewer moDerived macrophages in MTB-infected lungs, we analyzed recruited transitioning monocytes to understand if they behaved differently in the two infected environments. Compared to transitioning monocytes from uninfected mice, very few genes were differentially expressed in the same cell type from H37RvΔEccD_1_-infected mice (data S4 and fig. S4G). However, significant up-regulation was noted for several genes involved in MHC presentation from mice infected with H37Rv compared to H37RvΔEccD_1_ (Fig. 4, A and B). In addition, *Lars2*, which is important for protein synthesis (*40*), showed increased expression in transitioning monocytes from H37Rv-infected mice compared to those infected with H37RvΔEccD_1_.

To further understand ESX-1’s role within macrophages in the mouse lung, we directly compared H37Rv and H37RvΔEccD_1_-infected (i.e., zsGreen positive) moDerived macrophages using MAST (*34*). Notably, this cellular population was not detected in the uninfected mouse, and therefore, directly comparing cells not exposed to any bacilli was not feasible. By setting a log_2_ fold change threshold of 0.50, which indicates a 41% increase of gene expression level to enhance sensitivity, we identified eight significantly up-regulated genes in H37RvΔEccD_1_-infected moDerived macrophages: two predicted or long coding genes (*Gm10800* and *Gm15056*), IFN-inducible *Ifi27l2a*, as well as *Iqgap1*, *Dtx3l*, *Mtmr2*, *Cox17*, and *Sod1* (Fig. 4C). Among these, *Iqgap1* positively regulates autophagy and negatively modulates the type I IFN signaling pathway (*41*, *42*), suggesting a mechanism for better control of MTB. Conversely, in cells infected with H37Rv, up-regulation of 170 genes were noted (FDR < 0.05) including key genes involved in hypoxia-inducible factor–1 (HIF-1) signaling (*Hmox1*, *Nos2*, and *Eno1*), classical antigen presentation (*Cd74*, *H2-Q7*, and *H2-Dmb1*), and stimulation of cell death pathways (*Coro1a*, *Bax*, *NfKb2*, *Casp8*, and *Hmox1*) (Fig. 4D and data S5). QIAGEN Ingenuity Pathway Analysis (IPA) also revealed pathways favoring MTB survival (*2*), including alternative macrophage activation, C-X-C chemokine receptor type 4 (CXCR4) signaling, nuclear factor erythroid 2–related factor 2 (NRF2) oxidative response, necroptosis, and pyroptosis signaling (Fig. 4E). Gene ontology concurrently supported up-regulation of cellular locomotion and activation in H37Rv-infected cells (data S5). Together, the induction of permissive signatures in MTB-permissive macrophages, at least partially activated through multiple immune suppressive pathways (e.g., NRF2 oxidative response) (*43*, *44*), is ESX-1 dependent.

### ESX-1 suppresses the innate inflammatory response early in intracellular infection

**Fig. 5.**
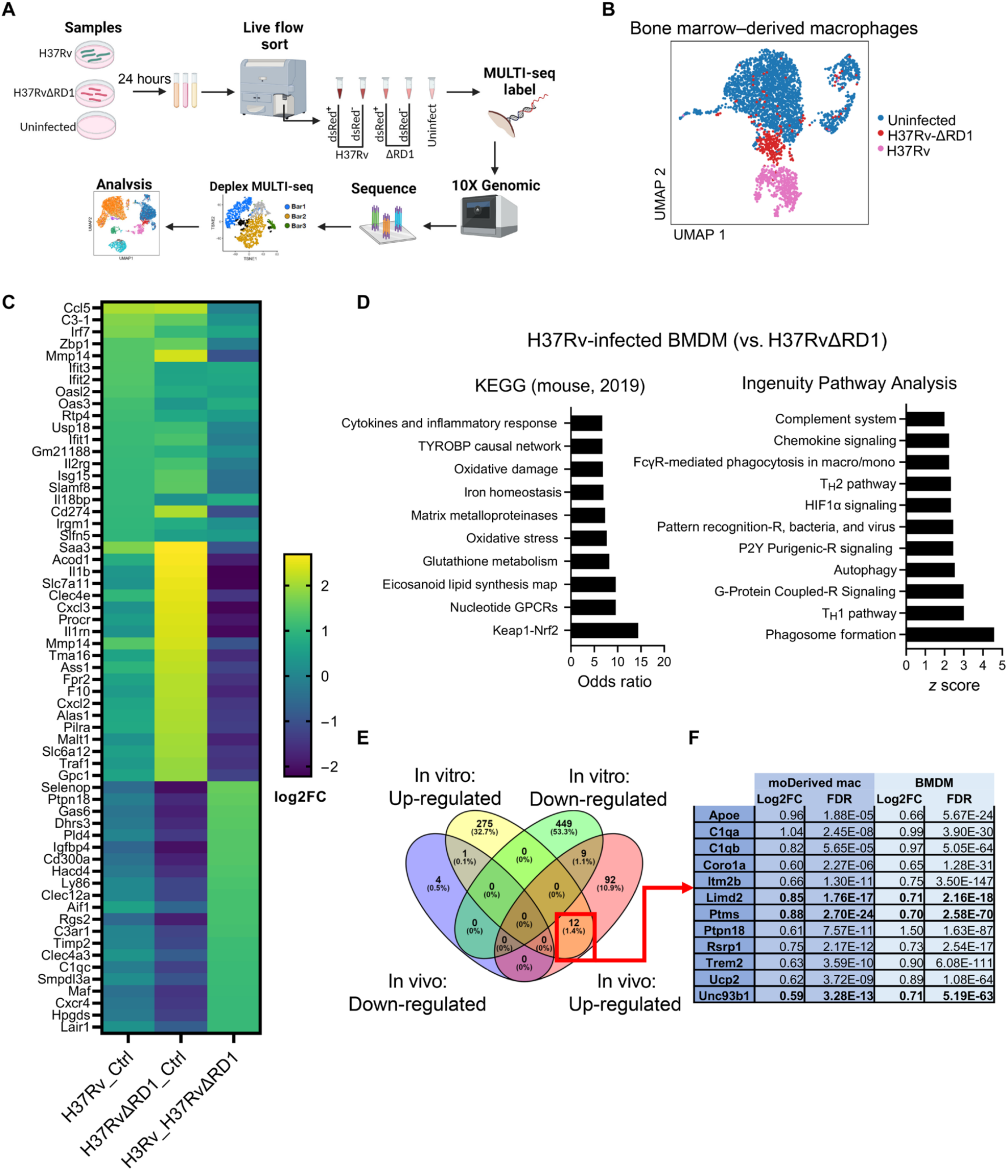
ESX-1 aids in the suppression of the inflammatory response in BMDM. (**A**) BMDM derived from C57BL/6 mice was infected with H37Rv or H37RvΔRD1 expressing dsRed for 24 hours, stained for viability, and flow-sorted on dsRed expression. Cells were multiplexed, and transcriptional libraries were obtained through 10X chemistry. (**B**) Dimension reduction of transcriptional libraries of each condition. (**C**) Heatmap of the top 20 differential gene expressions by log_2_ fold change of each comparison: H37Rv or H37RvΔRD1 versus control and H37Rv versus H37RvΔRD1. (**D**) Pathway enrichment of differentially expressed genes of BMDM infected with H37Rv as compared to H37RvΔRD1. (**E**) Comparing differentially expressed genes up or downregulated in ESX-1 active or inactive strains from in vivo (moDerived macrophages) or in vitro (BMDM) (**F**) revealed 12 genes concurrent in differential expression. Each condition was repeated in triplicate, both biologically and technically. FC, fold change

Bone marrow–derived macrophages (BMDMs), derived from bone marrow progenitors and differentiated in vitro, have been used as primary cells to study MTB infection. To compare with transcriptional differences identified in vivo, BMDM derived from healthy C57BL/6 mice were infected for 24 hours with MTB expressing dsRed with or without functional ESX-1 through lack of the entire RD1 operon (Fig. 2A); colony-forming unit (CFU) was similar in cells infected with both strains (fig. S5A). Live cells were sorted on dsRed expression, multiplexed using lipid-tagged indices (MULTI-seq), and single-cell transcriptomic libraries were produced using 10X chemistry (Fig. 5A and fig. S5B) (*45*).

Dimension reduction by principal component analysis followed by uniform manifold approximation and projection (UMAP) projection demonstrated a clear separation of H37Rv-infected BMDM from uninfected cells, with H37RvΔRD1 separated but more closely related to the uninfected population (Fig. 5B). Transcriptional clusters were noted, indicating heterogeneous responses despite the uniformity and control of the conditions. Overall, compared to cells not exposed to bacteria, both H37Rv- and H37RvΔRD1-infected cells showed a transcriptional up-regulation of cytokine signaling, oxidative stress, and necroptosis (Fig. 5C and data S6). In the setting of an infection period of 24 hours, there was an unexpected overlap in transcriptional changes between both infection states as compared to control. Nonetheless, differential regulation of key pathways was noted, such as ferroptosis and activation of the T helper 1 (T_H_1) pathway, which was significantly up-regulated with the presence of ESX-1 (fig. S5C).

To delve further into transcriptional differences between infection states, we directly compared BMDM infected with H37Rv with and without functional ESX-1. This short infection period did demonstrate the anticipated macrophage control of the intracellular bacteria through up-regulation of complement, *Aif1*, *Maf*, and pathway enrichment of oxidative stress, phagosome formation, and HIF-1α signaling. However, differential gene expression also revealed up-regulation of key genes associated with an anti-inflammatory response, such as *Selenop* (*46*), *Lair1* (*47*), and *Gas6* (*48*), with concurrent up-regulation of the NRF2 pathway when ESX-1 is present (Fig. 5, C and D).

Different environmental factors, cell types, methods of analysis, and time course of infection are but a few factors that have long accounted for the inability to translate findings from cells infected with MTB in vitro to those in vivo. By analyzing scRNA-seq data in our two models when ESX-1 function is lacking, we found that in both lung moDerived macrophage and BMDM, 12 genes were consistently differentially up-regulated in cells infected with H37Rv as compared to strains lacking functional ESX-1 (Fig. 5, E and F). Multiple genes up-regulated in both conditions have previously been demonstrated to play key roles in tuberculosis disease [*Apoe* (*49*)], immune evasion [*Trem2* (*50*)], inhibition of autophagosome formation [*Coro1a* (*51*)], biomarker discovery [*C1q* (*52*, *53*)], and general IFN-induced or inflammasome induction (*Itm2b* and *Ucp2*). Three novel genes—*Limd2*, *Ptms*, and *Unc93b1*—were also identified to correlate with MTB cellular infection. Together, ESX-1 induces immunoevasive responses in macrophages in vitro and in vivo, which is associated with successful MTB intracellular survival or replication.

### Activated and mature moDerived macrophages localize to the site of H37Rv bacilli

We identified heterogeneous cell types recruited to the lung 28 days after MTB infection. However, the spatial relationship between cells of each subset and bacilli cannot be determined with analysis of dissociated tissue. We therefore used Vizgen MERSCOPE Platform (MERFISH) (*54*), a spatial transcriptomics technique that combines highly multiplexed single-molecule fluorescence in situ hybridization (smFISH) and labeling of specific proteins to provide subcellular transcript location, cell type clustering, and spatial analysis of unique cells of interest (*55*). We further optimized specific lung processing to retain high-quality RNA and colabeled MTB with a polyclonal antibody to provide a single image acquisition of infected versus bystander cells (Methods and fig. S6).

**Fig. 6.**
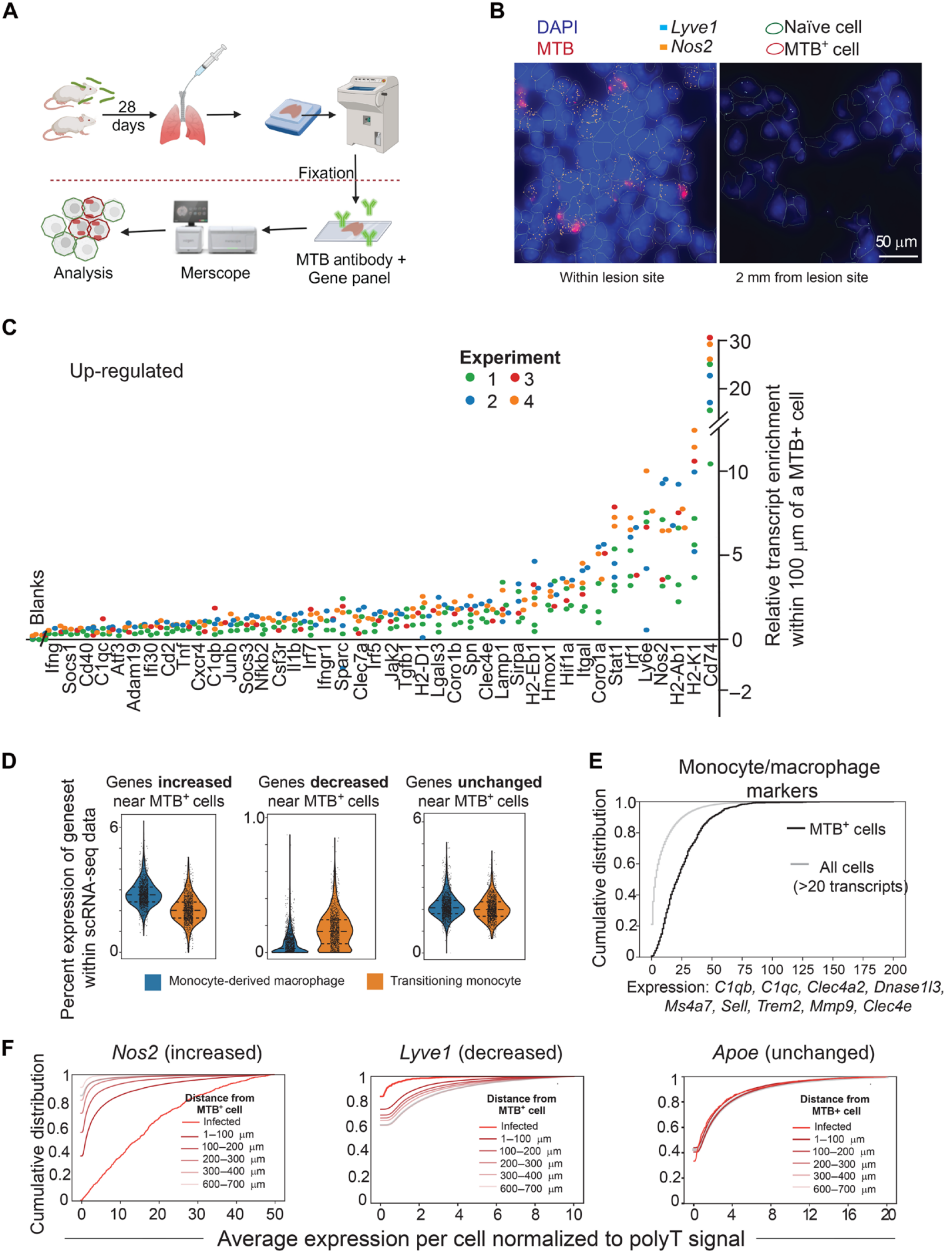
MTB induces maturation of macrophages only near its presence. (**A**) C57BL/6 mice were infected with H37Rv for 28 days, and after perfusion with PBS, lungs were inflated and freshly frozen in OCT. Slices (10 μm) were fixed and processed for Vizgen Merscope visualization of both MTB and gene panel. (**B**) Representative images from Merscope Visualizer. Red cell boundary indicates infected cells (i.e., MTB^+^), and green indicates uninfected cells (i.e., naïve). All circled cells express at least 20 transcripts from the panel. (**C**) Plot showing transcripts that are significantly up-regulated within 100 μm of MTB-infected cells. *y* axis is the difference between mean expression for a transcript in cells within 100 μm of MTB-infected cells as compared to that within all cells normalized to polyT (total mRNA per cell). All genes had a *P* value of <0.01 compared to pooled blank transcripts. (**D**) Violin plots showing the percent expression of up-regulated [shown in (C)], down-regulated (*Lyve1*, *Sup1*, *Scarf2*, *Itga6*, *Jun*, *Icam2*, and *Car4*), and unaffected genes within SmartSeq atlas shown in Fig. 4, with quartiles distinguished by lines within the violin plot. (**E**) Cumulative expression of representative markers for monocytes/macrophages. *x* axis represents the normalized gene counts from all listed MNP genes combined, and the *y* axis is the percentage of cells expressing less than or equal to the given expression level. A total of <1% of infected cells (black line) and <20% of all cells (gray line) have no detectable expression of the marker genes. (**F**) Representative cumulative distribution plots show a gene highly enriched near MTB-infected cells (*Nos2*), down-regulated near MTB-infected cells (*Lyve1*), and one that is unaffected by MTB presence (*Apoe*). Each color represents a bin of width 100 μm. Notably, not all lines start with *y* = 0 due to sparse gene expression levels. *N* = 3 uninfected, and *N* = 4 infected.

Lungs from C57BL/6 mice were infected with H37Rv for 28 days as in the previous experiments and labeled with a 122-gene panel constructed using gene signatures identified from our single-cell transcriptional atlas (Fig. 6A and table S3). We identified distinct cellular aggregates, previously described as non-necrotic lesions, further confirmed by MTB immunofluorescent staining (*56*). Using a high threshold to exclude background, infected cells were demarcated and transcripts were analyzed for proximity to infected cells within, as well as clusters outside, the lesions (Fig. 6B and fig. S6). Using data from eight distinct tissue sections from four different experiments, we identified 42 genes that were consistently up-regulated in cells within 100 μm of MTB-infected cells (*P* < 0.01 compared to blank probes; Fig. 6C). Transcripts concentrated within cells near those infected with H37Rv included *Hmox1*, *Nos2*, and *Hif1*α. Multiple IFN-induced genes were noted to increase toward MTB, such as *Irf1*, *Nfkb2*, *Il1b*, and *Ifi30.* However, the differentiation of type I and II could not be definitively made with the probe signature. In addition, the transcriptional up-regulation of MHC II antigen presentation increased toward MTB-infected cells (*Cd74*, *H2Ab1*, and *H2Eb1*) and an increase of *C1qb* and *C1qbc*, concordant with the signature of the mature moDerived macrophage seen in our transcriptional atlas. The moDerived macrophage gene signature was increased, and conversely transitioning monocyte signature decreased, in proximity to cells actively infected with MTB (Fig. 6D).

To ensure that these genes and others of interest were expressed by MNPs, we first confirmed that *Cd74* was expressed by more than 90% of cells. Secondarily, we used a gene signature that included nine canonical markers for monocytes and macrophages with no detectable expression in other mouse lung cells in a published single-cell dataset (*57*) (*C1qb*, *C1qc*, *Clec4a2*, *Dnase1l3*, Ms4a7, *Sell*, *Trem2*, *Mmp9*, and *Clec4e*) and found more than 80% of the cells analyzed expressed this signature, with the highest expression found in infected and neighboring cells (Fig. 6E and fig. S6C). Re-analyzing gene expression patterns in the population of strictly defined MNPs expressing detectable levels of these markers validated the previous up- and down-regulation of genes, including *Nos2* and *Lyve1* (Fig. 6F and fig. S6, D and E).

The gene signature down-regulated in neighbors of MTB-infected cells was less robust than the unregulated signature, with only seven genes consistently down-regulated. One of these genes, *Lyve1*, is expressed in both endothelial cells and perivascular anti-inflammatory macrophages. While the analysis shown here may include some endothelial cells, *Lyve1* was down-regulated even in strictly defined MNPs (fig. S6, D to F), in contrast to other anti-inflammatory genes including *Hmox1*, *Socs1*, and *Socs3*, which were consistently increased in the expression near sites of infection. Therefore, while cells with a permissive signature are recruited toward infected macrophages, they do not appear to be the *Lyve1*-high cells described elsewhere (*58*).

We asked what signature difference occurs between an infected macrophage and that of a nearby comparable cell type but uninfected (i.e. bystander). Only strictly defined monocyte/macrophage with at least five counts of markers were analyzed to compare similar populations. *Nos2* was the most significantly up-regulated gene in the infected versus bystander cells (fig. S7A). Further, genes related to MHC II expression were down-regulated in infected cells as previously described (*59*). Although limited by the gene panel, the differential expression in the spatial analysis correlated with comparison of infected to bystander moDerived macrophages and transitioning monocytes in the dissociated tissue analysis with significant up-regulation of *Nos2* and *Hmox1* (fig. S7B). Pathway prediction could not be completed due to the specificity of the gene panel, and therefore, the enrichment of key immune evasion genes warrants further evaluation. Overall, this technique supports the notion that mature moDerived macrophages were concentrated near the *Bacillus*-infected cells, with active differentiation near infected cells.

## DISCUSSION

Characterizing how MTB virulence factors alter host responses and cell recruitment to support MTB persistence is critical for understanding MTB pathogenesis and for identifying potential therapeutic targets. In this study, we demonstrate that after development of the adaptive immune response, the heterogeneity of recruited MNPs is significantly altered in the absence of ESX-1 function, and the induction of recruited MNPs with immune evasion signatures differs when ESX-1 is active. Further, spatial transcriptomics provides an understanding of macrophage activation at the level of individual cell infection. These results enhance our understanding of MTB’s ability to drive the maturation of macrophages for its benefit.

The diversity of lung myeloid cells remains incompletely understood during the later stages of MTB infection. Although ESX-1 is known to facilitate innate immune evasion by MTB and is essential for causing chronic infection, it remains unclear if it regulates lung myeloid cell heterogenicity and responses in vivo. Previously, we have described two major MNP subsets in tuberculosis infection through single-cell flow cytometry analysis (*8*, *9*, *12*, *16*). As anticipated based on recent evidence of macrophage heterogeneity (*35*, *60*), these cellular populations exhibit transcriptional heterogeneity. Our work extends recent findings of heterogeneous monocyte and macrophage recruitment during MTB infection at early infection to a period when the adaptive immune response is fully activated (*61*).

The ImmGen reference dataset was insufficient for refined cell identification in our comprehensive atlas, likely due to the complexity of novel cellular states during active infection compared to reference groups under homeostasis. While historical understanding of macrophage responses has relied on a dichotomous view of M1 and M2 (*62*), recent data demonstrate a more diverse spectrum (*35*, *63*, *64*). Our work aligns with this heterogeneity, and we therefore refrained from dichotomizing clusters to avoid oversimplification. Further, these findings extend from prior by showing that after the T cell activation, specific moDerived macrophages accumulate at a higher proportion in the presence of ESX-1. These moDerived macrophages harbor more bacteria and have a higher immune evasion signal compared to AMs, consistent with the previous finding that moDerived cells are MTB permissive during chronic infection (*12*). While differential recruitment may partially be explained by an overall lower bacterial burden and altered infection dynamics with an ESX-1 mutant (*6*, *12*, *22*), in the experiment for BMDM harboring a similar burden of wild-type or RD1 strain, scRNA-seq data demonstrated ESX-1–dependent responses. ESX-1 is known to regulate chemokine production (*20*), suggesting a regulatory role of ESX-1 in the recruitment of heterogeneous lung macrophages and the plasticity of macrophage responses in vivo during MTB infection.

The differential responses of macrophages both in vivo and in vitro provide further insight into the permissive host mechanisms related to MTB survival. Within both models, functional ESX-1 induces the expression of multiple immune evasion genes that are known to negatively regulate T cell activation, IFN-γ signaling, and phagosome maturation. For example, NRF2 signaling, considered to have anti-inflammatory defense through the expression of various antioxidants (*65*), is an important component of AM’s inability to control intracellular MTB (*2*). NRF2 also induces HMOX1 to inhibit NOS2-mediated nitric oxide production, which depends on ESAT-6 secreted by ESX-1 (*44*); inhibition of HMOX1 promotes T cell–mediated IFNγ/NOS2-dependent bacterial control (*66*). In addition, alternative activation pathways (e.g., interleukin-10) have been shown to not only inhibit the anti-MTB activities of macrophages but also suppress lung T cell activation (*67*, *68*), suggesting that these genes suppress both innate and adaptive responses to support MTB persistence in recruited macrophages during chronic infection. Recruited macrophages and BMDM also show induction of protective responses such as IFN signaling and the T_H_1 pathway. This observation holds in the spatial context, where cellular maturation and IFN stimulation occurred alongside the up-regulation of key protective factors. It is not clear whether the up-regulation of immune evasion genes is induced by prior inflammatory responses during MTB infection. Because these genes are known to hamper protective anti-MTB responses and enhance MTB survival, the induction of immune evasion responses may represent a virulence mechanism by MTB to impair antimycobacterial immunity to create a more permissive niche for replication. Together with previous findings (*19*, *20*), we hypothesize that ESX-1 assists in the recruitment of cells primed for manipulation and MTB persistence, and further work is needed to understand the signals required for differentiation of permissive MNPs in the MTB-infected lungs.

A key to comprehending the intricate interplay of MTB with recruited, infected, and bystander cells lies in elucidating spatial interactions. We have previously shown that Vizgen MERSCOPE, a microscopy-based spatial transcriptomic method (*54*), provides equivalent cell-type resolution and enhanced sensitivity compared to standard scRNA-seq methods (*55*). Using a panel of 122 genes, we identified spatial heterogenicity of cell types described in our 10X and SmartSeq2 atlas and unveiled genes that exhibit differential regulation across the myeloid microenvironment more comprehensively than previously reported (*69*, *70*). MHC II genes and Nos2 were the most significantly up-regulated transcripts toward the MTB-infected cell (*69*). Unexpectedly, we observed a reversal in the expression of *Lyve1*, although macrophages expressing high levels of Lyve1 have been implicated in an anti-inflammatory response and are associated with blood vessels in the lung (*58*). This discrepancy is likely due to the turnover of recruited cells and again highlights how chronic infection induces distinct macrophage responses that do not necessarily correlate with other inflammatory conditions. However, other markers of an anti-inflammatory response did increase toward MTB-infected cells, aligning with our findings that MTB infection prompts the recruitment of transcriptionally immunoevasive moDerived macrophages.

There are a few notable limitations to these experiments. Here, we used SmartSeq2, which, while highly effective, resulted in a restricted number of cells analyzed due to its inherent limitation in yield. However, the high-quality libraries and capability to capture rarer events of H37RvΔEccD_1_-infected mitigated this limitation. We found lower quality libraries in our 10X library than SmartSeq2 due to longer processing time from sort to load, increased fragility of cells after sort and washing, and the incapability to capture rarer events immediately at the sort (*71*). SmartSeq2 also provided the ability to capture discrete subsets such as the Notch-High subset, which harbors a high-fluorescent MTB burden similar to moDerived macrophages. Notch inhibitor reduces lung CFU and improves lung pathology in mice (*12*), suggesting a potential immunosuppressive role of Notch signaling in inhibiting host response to MTB infection. This work is largely transcriptional, and differential gene expression does not always correlate with significant differences in protein expression, warranting further investigation into expression variation among cellular subtypes. In addition, our study is limited to a specific time point post-development of the adaptive immune response, providing only a snapshot of the macrophage response to MTB without ESX-1, as the dynamics of infection are delayed during attenuated bacterial infection. Leveraging computational approaches, we believe our work can contribute and merge into the growing body of research using single-cell transcriptomics to elucidate the dynamics of recruited macrophage responses over time and enhance the understanding of MTB survival in host MNPs.

In summary, our study underscores ESX-1’s vital role in the recruitment of specific permissive macrophages with immunoevasive features and promoting macrophage maturation. Spatial transcriptional analysis provides a valuable avenue for understanding the in situ heterogeneity of gene expression in the infected microenvironment and offers further evidence of an induction of key permissive factors for bacterial survival. This work is also provided as a resource to the community to expand the host myeloid transcriptional plasticity during MTB infection over time and as a resource for comparing transcriptional atlases for a model of chronic infection.

## METHODS

### Study design

This work aimed to study the role of ESX-1 in MNP heterogeneity and response in mice and murine BMDMs using multiple methods of scRNA-seq. All experiments used randomly assigned mice. The number of biological replicates and the number of repetitions for each experiment are indicated in figure legends.

### Mice and care

C57BL/6 mice were purchased from the Jackson Laboratory and were between the ages of 6 to 8 weeks at the beginning of the experiment. For infections with MTB, mice were housed under barrier conditions in an animal BSL-3. Mice of both sexes were used. Mice were euthanized by CO_2_ asphyxiation followed by cervical dislocation. All experiments were performed with the prior approval of the University of California-San Francisco Institutional Animal Care and Use Committee (IACUC) under approval numbers AN180139-03 and AN194014-01.

### Bacterial strains and growth

All MTB strains were derived from an H37Rv background and grown as previously described (*12*). Constitutive fluorescent protein expression was obtained by transforming with pMSP12::mCherry, pMV261::ZsGreen, or pMSP12::dsRed2 and maintained by culturing in the presence of kanamycin (50 μg/ml). H37RvΔEccD_1_ mutant was constructed via abortive transduction as a method of allelic resistance substrate (AES) delivery (*27*). AESs were generated by flanking a hygromycin resistance cassette with ~500 base pair (bp) of *EccD_1_* (*Rv3877*). Transduced bacteria were selected on hygromycin-containing agar and confirmed through polymerase chain reaction (PCR), followed by Sanger sequencing of the region of interest.

### Aerosol infection

Bacterial strains were grown and stored for stock inoculum, with mice infected via the aerosol route using an inhalation exposure system (Glas-Col), as previously described (*6*, *8*, *9*, *12*, *16*). The target dose was 50 to 75 CFU per mouse for H37Rv and 500 CFU per mouse for H37RvDEccD_1_. The infectious dose was quantitated on day 1 by plating whole lung homogenates from three mice on Middlebrook 7H11 agar. The bacterial load at the time of analysis was also determined by serial dilutions plated on Middlebrook 7H11 agar. CFUs were counted after incubation of plates at 37°C for 3 weeks.

### Cell culture infection

BMDM were derived from bone marrow cells cultured in Dulbecco’s modified Eagle’s medium (Gibco, 11965092), 10% heat-inactivated fetal bovine serum (HI-FBS) and recombinant murine macrophage colony-stimulating factor (20 ng/ml; PeproTech) as described elsewhere (*6*). Before infection, cells were washed twice with phosphate-buffered saline (PBS). Bacterial strains were grown without tween and stored as previously described (*72*). Stock was thawed, and any remaining clumps were removed by filtering through a 5-mm filter. Cells were bathed in infection media at a multiplicity of infection of 1 for 4 hours, and washed, and media were replaced. Twenty-four hours after infection, live cells were lifted gently, washed, and stained by Zombie Aqua Fixable Viability Dye (BioLegend, 423101) before flow sorting.

### Lung homogenate preparation, flow cytometry, and cell sorting

Lung homogenates were prepared as previously described with modifications (*12*). For SmartSeq2 experiments, mice were retro-orbitally injected with anti–CD45-phycoerythrin for 2 min before euthanasia. For all experiments, after euthanasia, lungs were perfused with 10 ml of PBS/2 mM EDTA via the right ventricle immediately after euthanasia. Lungs were chopped into small pieces, placed in digestion media containing 4 ml of RPMI 1640/5% HI-FBS containing collagenase D (1 mg/ml; Sigma-Aldrich) and deoxyribonuclease I (30 μg/ml; Sigma-Aldrich), minced with a gentleMACS (Miltenyi, lung program1), and digested for 30 min at 37°C. Tissue was further minced with the gentleMACS (lung program2), passed through a 70-μm cell strainer, and rinsed, and red blood cells were lysed with 3 ml of ACK lysis buffer (Gibco) for 3 min prior and washed twice with RPMI 1640/5% HI-FBS.

Cells were stained with Zombie Aqua Fixable Viability Dye (BioLegend, 423101) for 15 min at 4°C, washed, and blocked at 1:100 with CD16/CD32 (BD, 553142) for 10 min before appropriate antibody staining for 30 min (table S4), diluted in Brilliant Stain Buffer (BD, 566349). Fluorescently labeled live cells were then acquired using a BSL3-contained Sony MAC900 cell sorter (figs. S1 and S5A). Acquisition data were analyzed using FlowJo software (TreeStar). To obtain MFI at time of cell sort, data were reviewed using the Sony MA900 software from sort of each run and using Boolean expression, tabulated for integration into the transcriptional data object.

### Single-cell cDNA generation, library construction, and sequencing

#### SmartSeq2

RNA transcriptomics were obtained from sorted single cells following SmartSeq2 protocol from Picelli *et al*. (*24*) using high-throughput liquid dispensers detailed elsewhere, which includes: preparation of lysis plates; cDNA synthesis, library preparation using Illumina Nextera XT kit, library pool, and ampure bead clean-up; and quality control. To provide feasibility within a BSL3, plates containing reverse transcriptase were modified to provide centrifugation for distribution. For further details, please refer to https://dx.doi.org/10.17504/protocols.io.8epv5xx54g1b/v1. Library pools were sequenced on the NovaSeq 6000 Sequencing System (Illumina) using 2 × 100-bp paired-end reads and 2 × 12-bp index reads with a 300-cycle kit (Illumina, 20012860).

#### 10X Single Cell 3′ GEM

For lung myeloid cells, single cells were sorted as described, washed, and manually counted with trypan blue on a hemocytometer. GEMS (gell beads-in-emulsions) were generated using a 3′ v3.1 kit according to the manufacturer’s protocol on a 10X Genomics Chip A without multiplexing, with each condition loaded on 2 separate lanes. For in vitro BMDM, cells were labeled using the MultiSeq labeling method as detailed further in https://doi.org/10.1038/s41592-019-0433-8 before using a 3′ v3.1 kit and loading. Cleanup and cDNA amplification were done following the manufacturer’s protocol.

#### Library construction and sequencing

Libraries were constructed using the Chromium Next GEM Single Cell 3’ Library Kit v3.1 without deviation, yields assessed using Agilent TapeStation, and quantified via quantitative PCR (KAPA Library Quantification Kits for Illumina platforms, KK4828). Plate pools were normalized, combined at an equimolar ratio of 2 nM, and sequenced on a NovaSeq 6000 Sequencing System (Illumina) using 28 + 91–bp paired-end reads and 8-bp Index 1 reads with a 100-cycle kit (Illumina, 20028400).

### Data extraction and analysis

#### Alignment

Sequences from the NovaSeq were demultiplexed using bcl2fastq version 2.20. Sequenced reads from SmartSeq2 libraries were aligned with Gencode v.M19 annotations and Gencode v.M19.ERCC using STAR version 2.5.2b with parameters TK. Sequenced reads from 10x Single Cell 3′ libraries were aligned with a custom mouse reference genome with added mCherry sequences that was created using CellRanger v.5.0.1 software.

#### Dimension reduction and differential gene expression

After standard preprocessing, dimension reduction was conducted using Scanpy (Python). Objects were converted to R for differential gene expression (DE) using MAST. Significantly expressed genes (FDR < 0.05) were used for Gene Ontology (ShinyGOv0.61 and EnrichR). IPA (Qiagen) was used on genes with FDR < 0.1 and ranked by log2 fold change to identify regulation direction of pathways.

#### Gene enrichment scores

Enrichment scores were calculated from top enriched pathways and previously described macrophage transcriptional phenotypes using Scanpy (table S2) (*35*, *62*).

### Spatial transcriptomics

C57BL/6 mice were aerosol infected with H37Rv-zsGreen at ~75 CFU per mouse for 28 days. Lungs were perfused with 10 ml of PBS/2 mM EDTA via the right ventricle, followed by inflation through the trachea with 1 ml of optimal cutting temperature (OCT) mixed with PBS at a 1:1 ratio. Lungs were embedded in OCT, flash-frozen, and stored at −80°C. To confirm quality, RNA integrity number (RIN) was obtained by extracting RNA from each block (QIAGEN RNeasy Mini Kit, catalog no. 74104) and quantity analyzed on an Agilent Tapestation system. OCT-embedded tissue blocks were cryosectioned at −20°C to a thickness of 10 μm, mounted onto MERSCOPE Slides (Vizgen, PN 20400001), and immediately fixed with 4% paraformaldehyde at 37°C for 30 min. Slides were washed with 3x PBS and stored in 70% ethanol at 4°C for no more than 1 month before proceeding following the manufacturer’s protocol (MERSCOPE) as previously described (*55*) with the following exceptions. First, cell boundaries were stained with the Vizgen Cell boundary Staining Kit (catalog no. 10400009) in conjunction with a polyclonal MTB antibody (Abcam, ab905) raised in rabbit diluted to 1:100. After washing, the Secondary Staining Mix (Vizgen, PN 20300011) was diluted with a blocking solution in two-step dilution (first, 1:100; second, 1:33) to ensure correct ratio of primary to secondary staining because the primary MTB antibody was used.

#### Imaging

The MERFISH gene panel consisted of 122 genes, designed by selecting top differentially expressed genes from the myeloid scRNA atlas, canonical markers, and genes of interest (table S3). The probes were hybridized; samples washed before gel embedding, treated with a clearing solution, and 1 day later, added 4′,6-diamidino-2-phenylindole (DAPI)/PolyT Staining Reagent added, and imaged per MERSCOPE User Guide 91600001.

#### Image analysis

The full code can be found here https://github.com/bspeco/ESX1_macrophageheterogeneity_scRNA/blob/main/TBSpatial_Analysis_code.ipynb

All figures represent two mice analyzed in four separate experiments, each measuring one to three lung sections. “Replicate” refers to an individual section. Results were highly concordant whether considering separate sections, experiments, mice, or the sum total.

For the spatial analysis showing differential abundance of transcripts near MTB-positive cells, the Vizgen cell by gene matrix was first filtered to include cells with at least 20 transcripts and with a volume between 200 and 5000 μm³ with a cumulative DAPI signal greater than 2 million. Areas of tissue that showed evidence of damage (i.e., tissue folding or tearing) were excluded from downstream analysis. MTB-positive cells were defined as any cell with top 100 brightest pixels in the anti-MTB channel averaged above 300 units. Cells with an SD in this channel lower than 150 (i.e., cells with uniform brightness) or further than 400 μm from their two nearest neighbors (i.e., isolated in space) were not counted. This threshold was chosen by picking a range of values, labeling the resulting positive cells in the Merscope Visualizer app, and checking for accurate labels compared to the MTB antibody channel. The chosen thresholds limited the selection of clearly negative cells while positively labeling the majority of cells with three or more MTB puncta.

The euclidean distance from each cell in the tissue to its nearest MTB positively stained neighbor was calculated. Transcripts enriched near MTB-positive cells were identified by binning the cells found within 100 μm (~5 cell widths) of a MTB-positive cell (excluding those infected), and average transcript expression was compared to that in all cells 100 μm or further from the MTB-positive cell. To accomplish this, each cell’s transcripts were normalized to the total RNA content of the cell by dividing the transcript counts by the cellular polyT signal and multiplying by the average polyT signal for that section. Expression levels for each transcript were then compared within each section using a bootstrap permutation test (run 10,000×) comparing the actual difference in means to the difference in means when the cell location labels are randomly distributed. Transcripts with a mean increase or decrease in average count near MTB-positive cells that were greater than the expected (random) distribution in at least 9990 of 10,000 permutations (*P* < 0.001) were counted as significant. The measurements for all replicates were combined, and a Kruskal-Wallis test was run to find transcripts whose enrichment over all the replicates differed from the background (Blank) signal. The violin plots were generated by selecting genes that were consistently differentially regulated across all replicates (Kruskal-Wallis test versus all blanks, *P* < 0.01). Genes were grouped into an “up-regulated,” “downregulated,” or “neither” geneset, and their total percent expression was calculated for each cell in the scRNA-seq SmartSeq2 dataset.

### Statistical analysis

Flow cytometry experiments were performed with five mice per condition in at least three separate experiments. Results are expressed as mean and SD. To assess the distribution of data, Kolmogorov-Smirnov test was performed on each condition. On the basis of these results, nonparametric tests were used. The Holm-Sidak method was applied for multiple comparisons to control the family-wise error rate. Statistical significance was set at a 95% confidence interval with *P* < 0.05 considered significant. Detailed statistical approaches for gene expression and pathway analysis are provided above.
