# Supplementary material for: Single-cell analysis reveals Mycobacterium tuberculosis ESX-1–mediated accumulation of permissive macrophages in infected mouse lungs
Source: Sci Adv. 2025 Jan 15;11(3):eadq8158. doi: 10.1126/sciadv.adq8158 (PMC11734715; doi:10.1126/sciadv.adq8158)
Supplement: Supplementary file 1 — Figs. S1 to S7 Tables S1 to S4 Legends for data S1 to S6 [file sciadv.adq8158_sm.pdf]

Science Advances
AAAS

Supplementary Materials for

Single-cell analysis reveals *Mycobacterium tuberculosis* ESX-1–mediated accumulation of permissive macrophages in infected mouse lungs

Weihao Zheng *et al.*

Corresponding author: Beth Shoshana Zha, shoshana.zha@ucsf.edu

Sci. Adv. **11**, eadq8158 (2025)
DOI: 10.1126/sciadv.adq8158

The PDF file includes:

Figs. S1 to S7
Tables S1 to S4
Legends for data S1 to S6

Other Supplementary Material for this manuscript includes the following:

Data S1 to S6

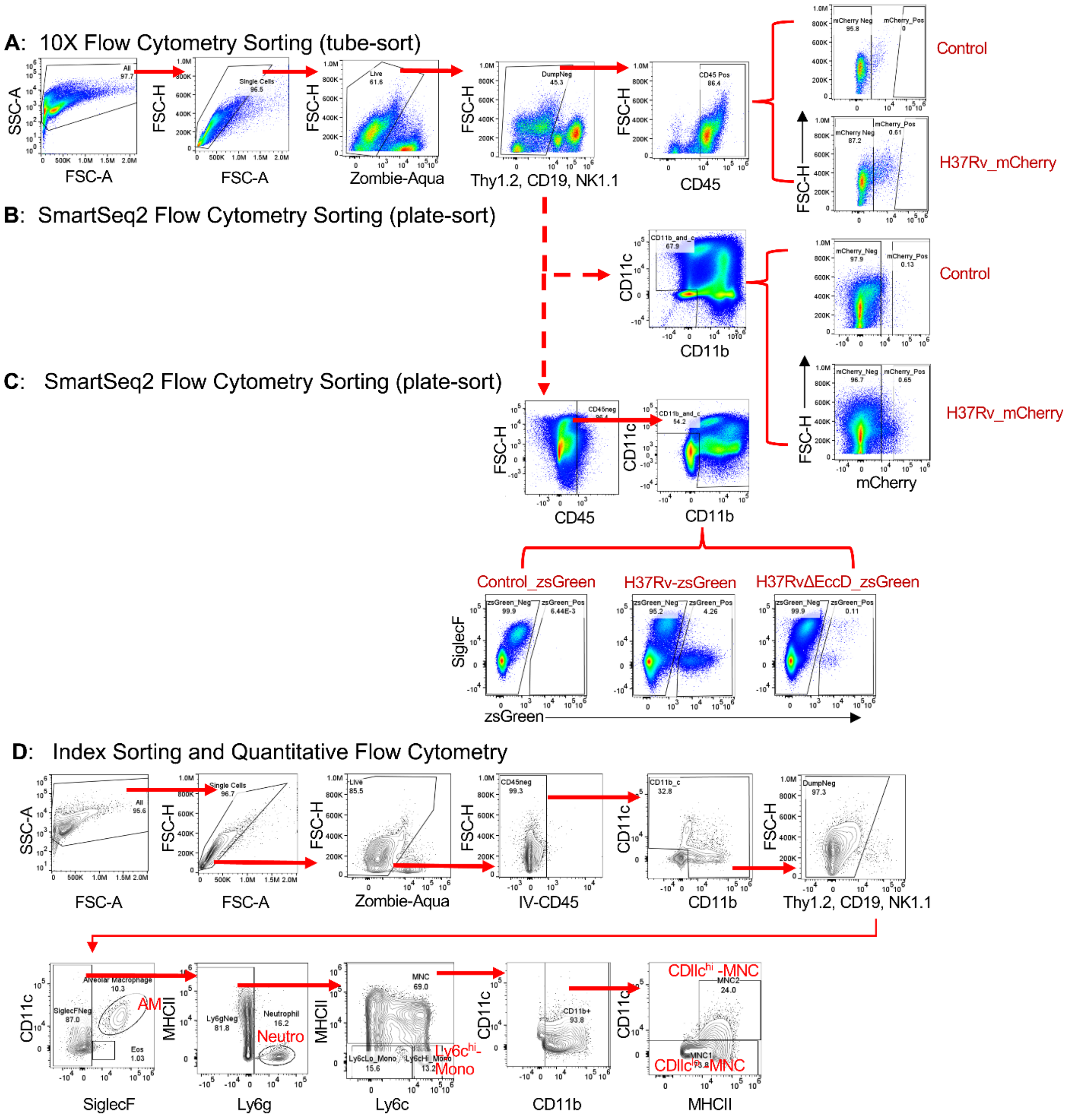

Fig. S1. Flow cytometry sorting scheme to obtain lung mononuclear phagocytes in mice infected with MTB. (**A**) After gating out NK, T, and B cells, live single CD45+ cells were sorted into separate tubes based on mCherry positivity and subjected to 10X Chromium loading. **(B)** Cells were sorted on CD11c and/or CD11b positivity for a more targeted analysis, with mCherry positive or negative sorted separately into 384-well plates. **(C)** In the setting of H37RvΔ$EccD_1$ infection, zsGreen fluorescent MTB protein expression was utilized for improved demarcations. Intravascular cells were further assured to be removed through the addition of intravascular CD45 antibody. **(D)** Gating strategy for quantitative flow cytometry and for index sorting analysis used in panels B and C. Cells were divided into alveolar macrophages (AM; $CD11b^{lo}CD11c^{hi}SiglecF^{hi}$), neutrophil (neutro; $SiglecF^{-}Ly6G^{hi}CD11b^{hi}$), $CD11c^{lo}$-mononuclear cell (MNC; $CD11b^{+}CD11c^{lo}MHCII^{+}$) and $CD11c^{hi}$-mononuclear cell (MNC2; $CD11b^{+}CD11c^{hi}MHCII^{hi}$). Further analysis based on MTB-fluorescent protein expression was also completed. For all experiments, lung perfusion with PBS-EDTA was performed prior to processing.

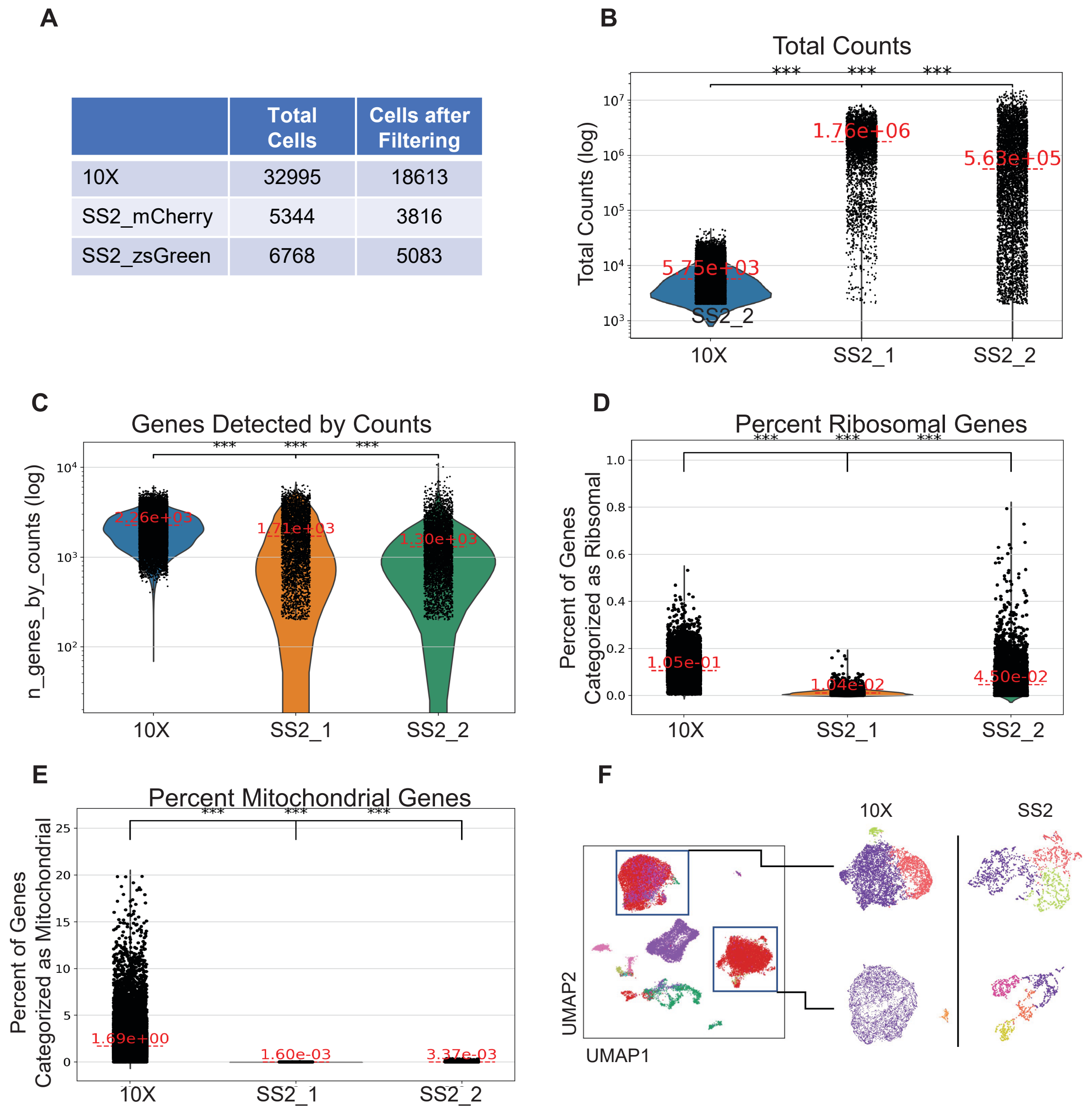

	Total Cells	Cells after Filtering
10X	32995	18613
SS2_mCherry	5344	3816
SS2_zsGreen	6768	5083

Fig. S2. SmartSeq2 provides improved library depth when analyzing lung mononuclear phagocytes in mice infected with MTB. **(A)** Cell counts from the three single-cell experiments. (**B**) Total counts, **(C)** genes (as normalized by counts), **(D)** ribosomal counts, and **(E)** mitochondrial counts for each condition, where ***p<0.001. **(F)** Using cellxgene, the two macrophage clusters were extracted by experimental methods (i.e., 10X or smartseq2) and clustered using Leiden at a resolution of 0.2 (differential color delineation is per subcluster).

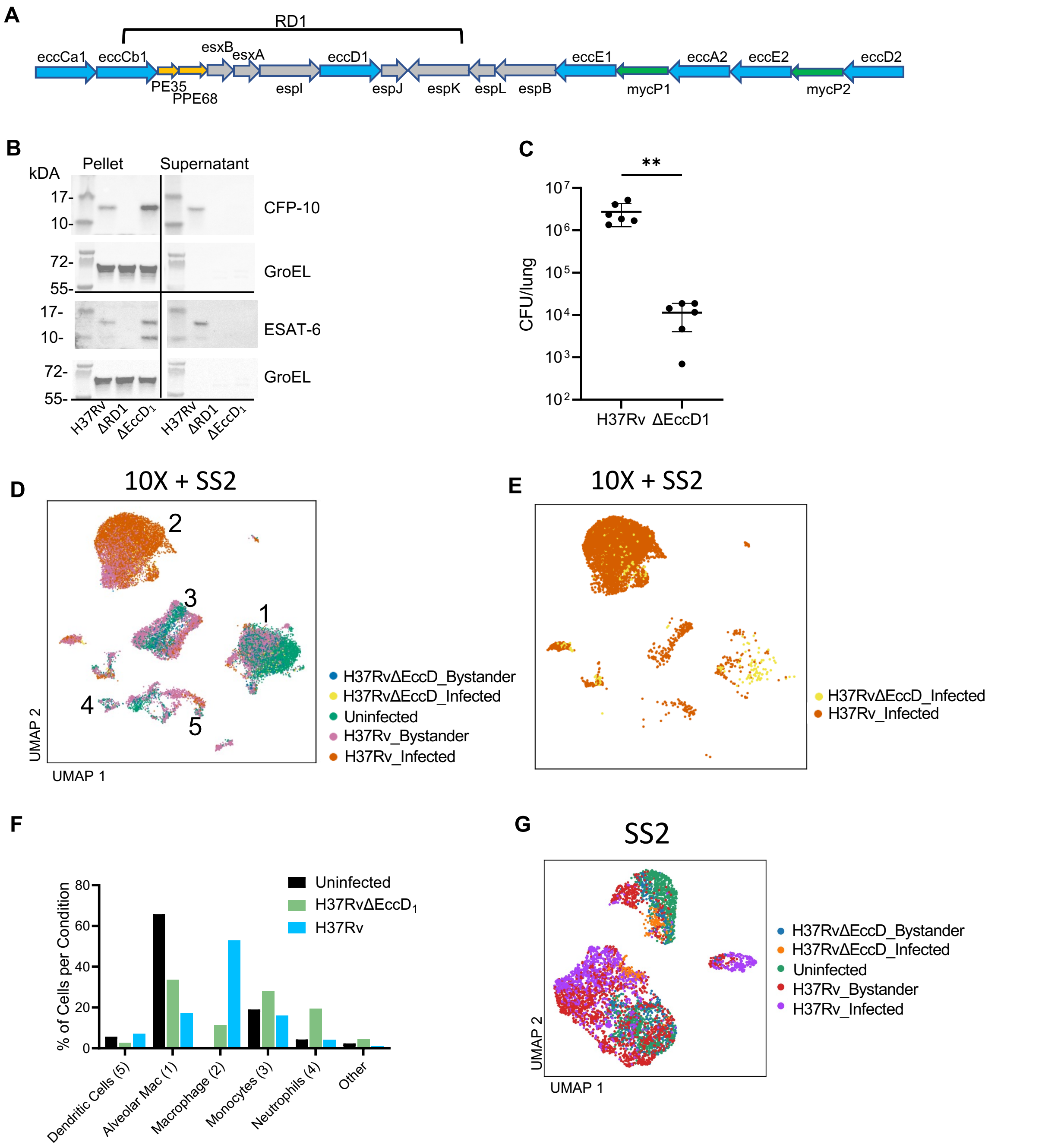

Fig. S3. Macrophages are recruited differentially with or without ESX-1 presence. **(A)** ESX-1 is a multi-protein complex encoded throughout, as well as outside, the RD1 operon. The RD1 operon also encompasses other genes not related to the ESX-1 complex. **(B)** Representative immunoblots were used to evaluate ESX-1-dependent CFP-10 and ESAT-6 secretion and expression in H37Rv, H37RvΔRD1, and H37RvΔ$EccD_1$ strains. **(C)** Colony forming units (CFU) from lungs of mice 28 days after aerosol infection with H37Rv or H37RvΔ$EccD_1$, where ** denotes $p<0.01$. **(D)** Dimensionally reduced integrated data from 3 separate single-cell experiments depicts infected states, where 'infected' was determined by MTB-fluorescent protein expression, 'bystander' cells not expressing MTB-fluorescent protein, and 'uninfected' cells from mice not exposed to MTB. **(E)** Copy of Panel D to only include cells infected with H37Rv or H37RvΔ$EccD_1$ for improved visualization. **(F)** Quantification of cell types recruited per condition of total atlas (10X + SS2), with percentage representative of that condition (ex. % macrophages over all cells in H37Rv-infected mice). Cell types are derived from panel C. Total cell numbers can be found in Table S1. **(G)** Removal of 10X dataset with re-clustering of mononuclear cells obtained from SmartSeq2 (Scanpy).

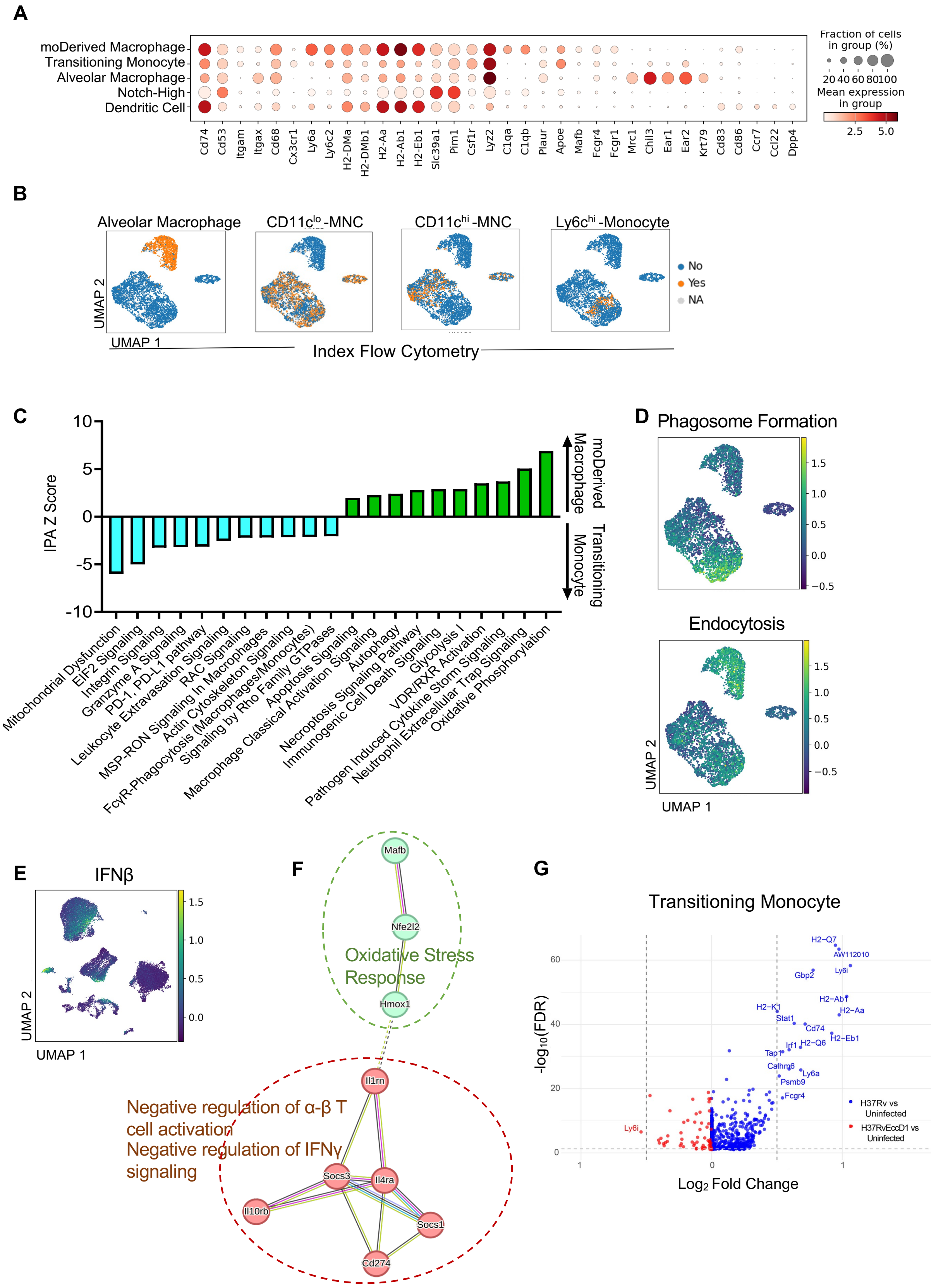
A
moDerived Macrophage
Transitioning Monocyte
Alveolar Macrophage
Notch-High
Dendritic Cell
Cd74 Cd53 Itgam Itgax Cd68 Cx3cr1 Ly6a Ly6c2 H2-DMa H2-DMb1 H2-Aa H2-Ab1 H2-Eb1 Slc39a1 Pim1 Csf1r Lyz2 C1qa C1qb Plaur Apoe Mafb Fcgr4 Fcgr1 Mrc1 Chil3 Ear1 Ear2 Krt79 Cd83 Cd86 Ccr7 Ccl22 Dpp4
Fraction of cells in group (%)
20 40 60 80 100
Mean expression in group
2.5 5.0
B
Alveolar Macrophage
CD11clo-MNC
CD11chi-MNC
Ly6chi-Monocyte
No
Yes
NA
UMAP 2
UMAP 1
Index Flow Cytometry
C
IPA Z Score
10
5
0
-5
-10
moDerived Macrophage
Transitioning Monocyte
Mitochondrial Dysfunction
EIF2 Signaling
Integrin Signaling
Granzyme A Signaling
PD-1, PD-L1 pathway
Leukocyte Extravasation Signaling
RAC Signaling
MSP-RON Signaling In Macrophages
Actin Cytoskeleton Signaling
FcγR-Phagocytosis (Macrophages/Monocytes)
Signaling by Rho Family GTPases
Apoptosis Signaling
Macrophage Classical Activation Signaling
Autophagy
Necroptosis Signaling Pathway
Immunogenic Cell Death Signaling
Glycolysis I
VDR/RXR Activation
Pathogen Induced Cytokine Storm Signaling
Neutrophil Extracellular Trap Signaling
Oxidative Phosphorylation
D
Phagosome Formation
Endocytosis
1.5
1.0
0.5
0.0
−0.5
UMAP 2
UMAP 1
E
IFNβ
1.5
1.0
0.5
0.0
UMAP 2
UMAP 1
F
Mafb
Nfe2l2
Hmox1
Oxidative Stress Response
Il1rn
Socs3
Il4ra
Il10rb
Socs1
Cd274
Negative regulation of α-β T cell activation
Negative regulation of IFNγ signaling
G
Transitioning Monocyte
-log10(FDR)
60
40
20
0
Log2 Fold Change
1
0
1
H2−Q7
AW112010
Ly6i
Gbp2
H2−Ab1
H2−Aa
H2−K1
Stat1
Cd74
H2−Eb1
H2−Q6
Irf1
Tap1
Calhm6
Ly6a
Psmb9
Fcgr4
Ly6i
H37Rv vs Uninfected
H37RvEccD1 vs Uninfected

Fig. S4. MTB induces recruitment of two large monocyte-derived cell subsets with differential responses to H37Rv and H37Rv lacking functional ESX-1. C57BL/6 mice were infected with H37Rv or H37RvΔEccD$_1$ for 28 days. Single-cell suspensions were sorted and processed using SmartSeq2 (Figure 2). **(A)** Transcriptional canonical markers confirm subset delineation naming. **(B)** Cell subpopulations identified by index sorting are overlaid with cell types identified by single-cell transcriptional clusters. **(C)** Differential gene expression was conducted using MAST to compare recruited transitioning monocyte and monocyte-derived macrophages. Those with FDR <0.05 were used for Ingenuity Pathway Analysis. Shown are pathways with FDR <0.05 and removal of redundancy. (**D)** Gene sets were constructed to score the likelihood of a particular cell in the SS2 atlas with transcriptional enrichment of the denoted pathway using the average expression of associated genes (methods). The lighter color denotes the higher expression of the gene set and thus predicts the attribute. **(E)** Enrichment using gene set for IFNβ overlaid on the parent atlas (10X + SS2). **(F)** STRING multi-cluster engine analysis of immune evasion proteins using default parameters. (**G**) Volcano plot of transitioning monocytes comparing upregulated genes from bystander cells isolated from H37Rv-infected mice compared to uninfected mice (blue) and upregulated genes from bystander cells from H37RvΔEccD$_1$-infected mice compared to uninfected mice.

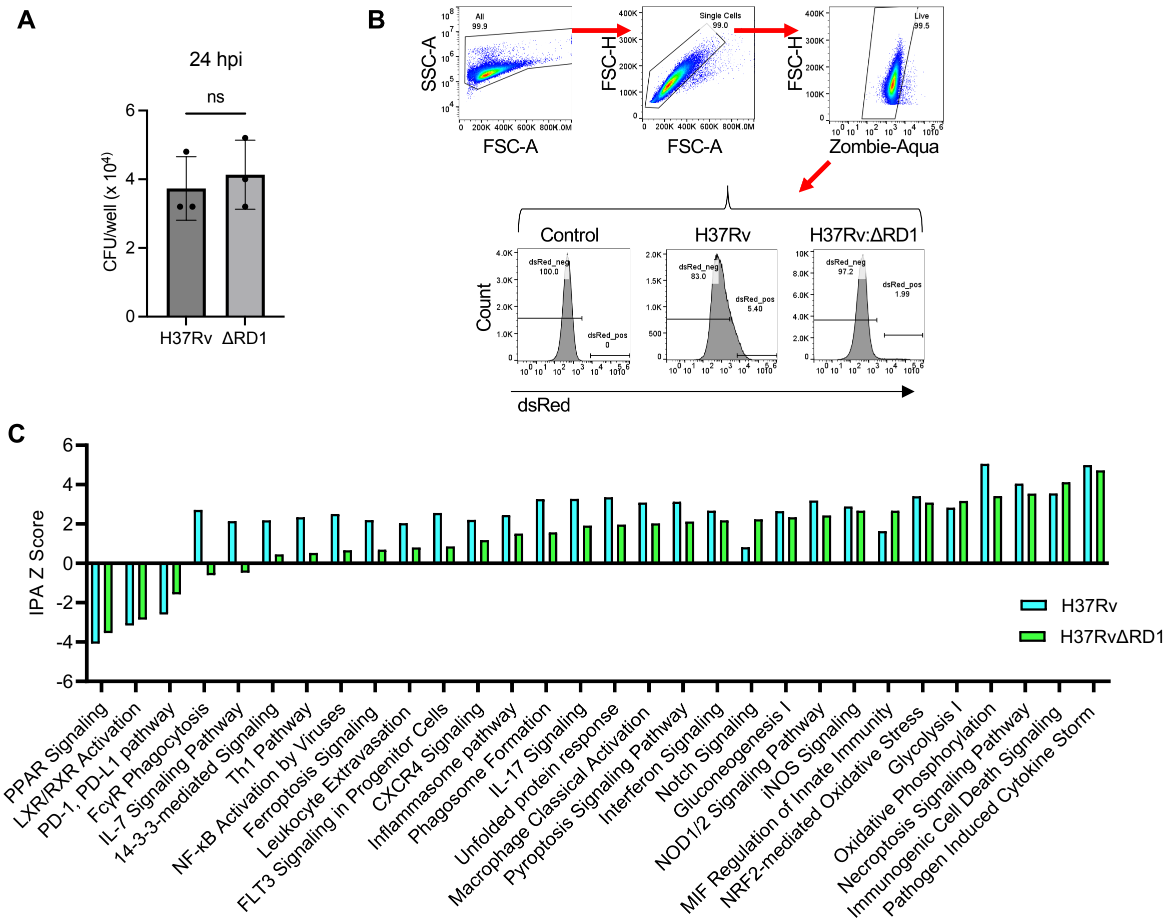

Fig. S5. BMDM infected with H37Rv and H37RvΔRD1 have similar CFU at 24h but differential transcriptional responses. (A) CFU for BMDM infected with H37Rv or H37RvΔRD1 for 24h. **(B)** Flow cytometry sorting scheme of BMDM infected with H37Rv-dsRed, H37RvΔRD1-dsRed, or uninfected to obtain transcriptional libraries. After 24h of infection, cells were collected, washed, and stained with zombie-aqua for live/dead delineation, filtered into a single cell suspension, and sorted in a BSL3 contained Sony MA900. Shown are representative images of single live cells, with cutoffs for dsRed positivity to delineate infected from bystander/uninfected cells. **(C)** Qiagen Ingenuity pathway analysis was queried using differentially expressed genes determined by MAST analysis of infected cells compared to control, with an FDR <0.05. Shown are significantly enriched pathways scaled on Z score for directionality analysis, with >|2| considered significant.

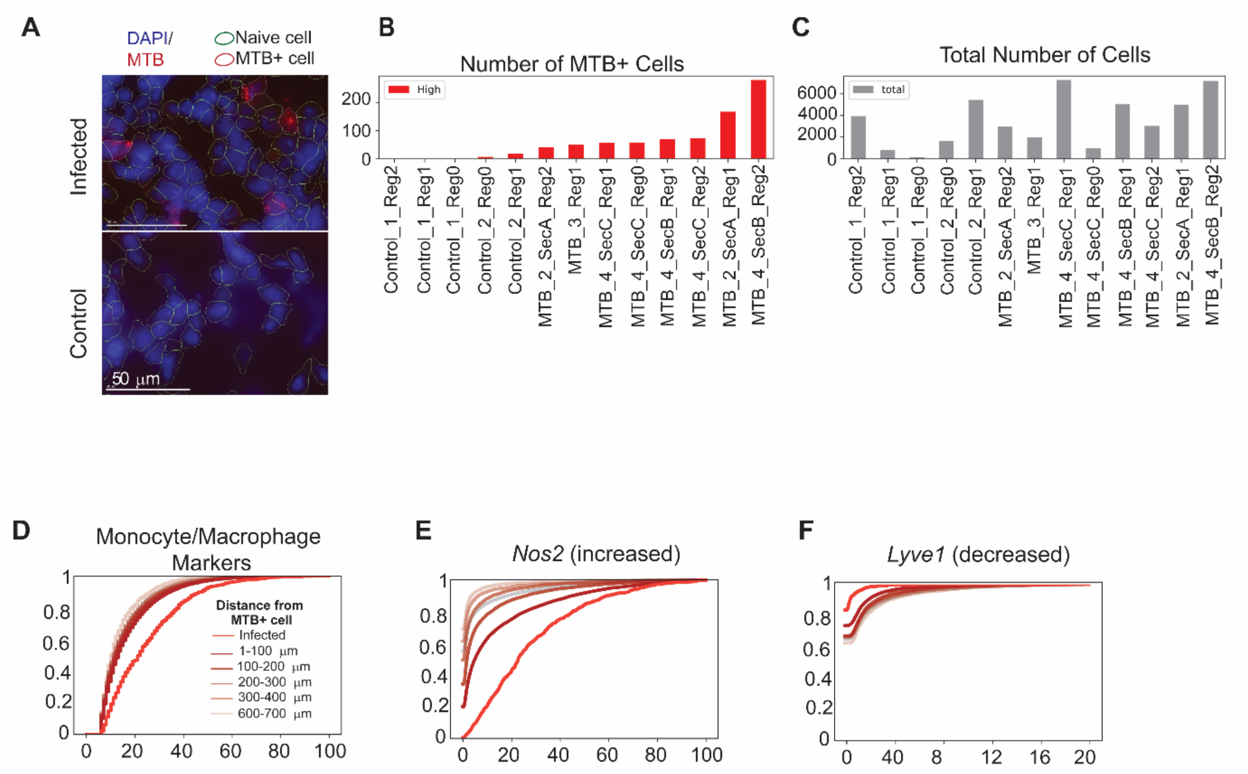

Fig. S6. Quality control for Vizgen Merscope spatial transcriptomics. **(A)** Identification of MTB positively infected cells in tissue slices obtained from control (uninfected) and H37Rv-infected C57BL/6 mice. MTB-infected cells were identified by averaging the brightest 100 pixels from Cellbound3 (MTB antibody) channel per cell. Any cell in which this average was 300 or greater with a standard deviation in the MTB antibody channel greater than 150 was labeled as infected. Cells with lower standard deviations represented bright circular artifact staining and were not counted. Any cells that were outliers in space (lacking two or more infected neighbors within half a millimeter) or in brightness (>5 million average cellbound3 intensity) were also removed. Regions with obvious tissue defects that produced large artifacts in the cellbound3 channel (i.e. tears) were excluded. **(B)** Number of MTB-infected cells in tissue slices, with **(C)** total cell counts per section for comparison. **(D)** Cumulative distribution plot of strictly defined monocyte/macrophages (*C1qb, C1qc, Clec4a2, Dnase1l3, Ms4a7, Sell, Trem2, Mmp9, Clec43*). In addition, cumulative distribution plots for **(E)** *Nos2* and **(F)** *Lyve1* within strictly defined monocyte/macrophages show overall concordance with Fig. 6.

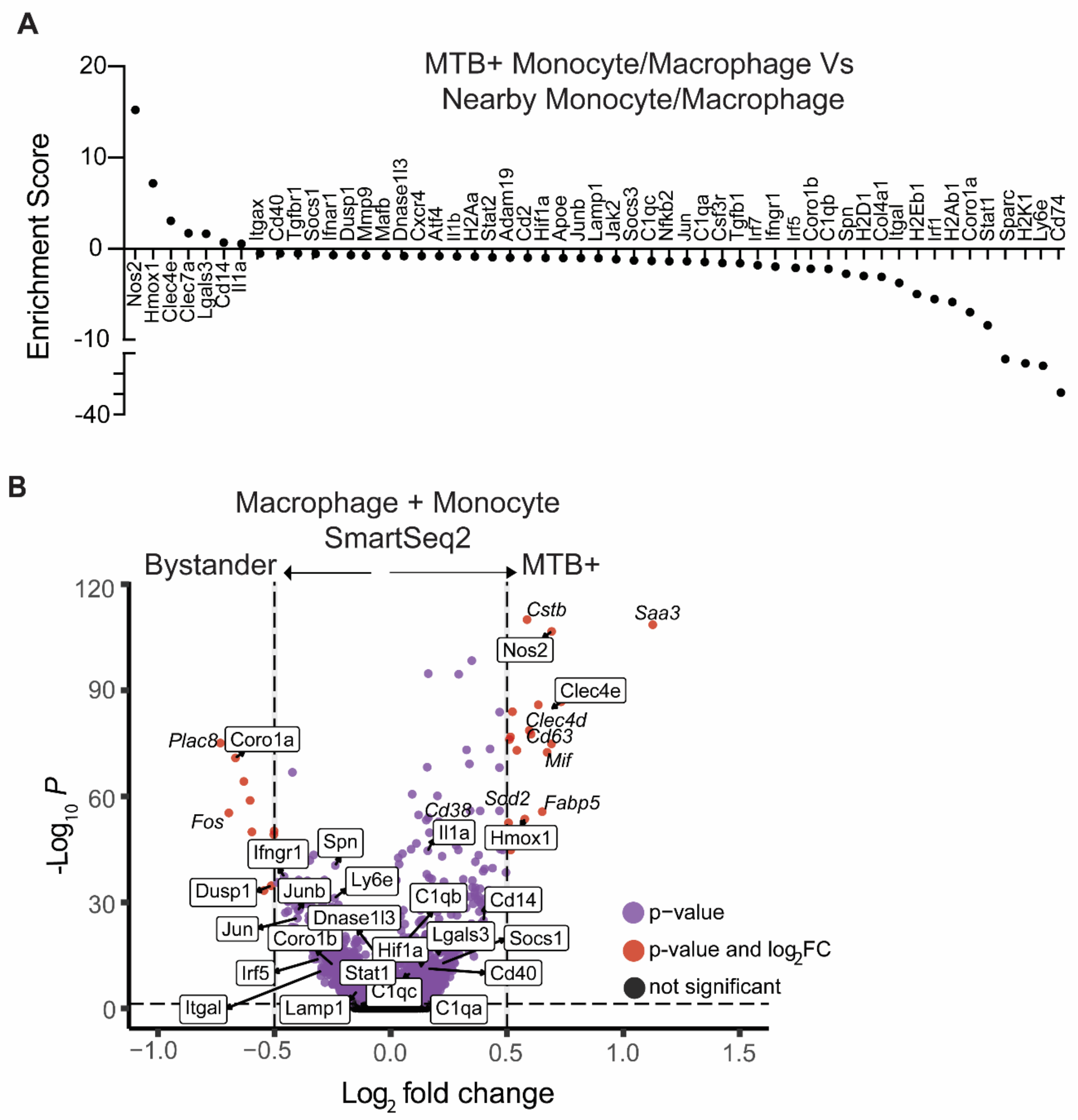

Fig. S7. Comparison of gene expression between MTB-infected and bystander cells from spatial and SmartSeq2 datasets. (A) Gene enrichment score (difference in average normalized gene expression) between infected monocytes and macrophages compared to neighboring (within 100 µm) uninfected cells. All cells included in the analysis expressed five or more transcripts from a list of canonical markers not expressed in other cell types (*C1qb, C1qc, Clec4a2, Dnase1l3, Ms4a7, Sell, Trem2, Mmp9, Clec4e*). All genes displayed had a p-value of <0.001 based on a permutation test with 1000 iterations. **(B)** Differential gene expression of moDerived macrophages and transitioning monocytes from SmartSeq2 by MAST, comparing cells expressing MTB fluorophore (MTB+) versus those not expressing fluorophore (bystander) from H37Rv-infected mice. Genes from panel A are boxed for visualization.

Table S1: Final cell numbers and proportions. After quality control, mononuclear cells obtained for SmartSeq2 were analyzed for proportion in each condition (total obtained in FigS2A). Statistical analysis was conducted using a 2-sample chi-square with Yates' continuity correction).

Cell Type	Uninfected (% Total)	H37RvΔEccD$_1$ (% Total)	H37Rv (% Total)	H37Rv versus H37RvΔEccD$_1$ (p-Val)
**moDerived Macrophage**	0 (0)	83 (11.6)	1656 (54.01)	<0.0001
**Transitional Monocyte**	524 (43.49)	289 (40.45)	683 (22.28)	<0.0001
**Alveolar Macrophage**	610 (50.62)	297 (41.6)	295 (9.62)	<0.0001
**Notch^{Hi}**	0 (0)	6 (0.84)	273 (8.9)	<0.0001
**Dendritic Cell**	71 (5.89)	39 (5.46)	159 (5.19)	NS (0.85)

Table S2: Genes utilized for each score name (Figure 3). *Denotes scores that were exhibited in *Sanin et al* (*35*).

Gene Set Name	
Anti-Inflammatory	*Cd274, Hmox1, Il4ra, Il10rb, Il1rn, Mafb, Nfe2l2, Socs3, Socs1*
MHC Class II complex*	*H2-Ab1, H2-Aa, H2-Eb1, H2-Oa, H2-DMb2, H2-Ob, H2-DMb1*
Endocytosis	*Apoe, Trf, Trem2, Cd36, Snx1, Cd9, Anxa2, Trem2, Arrb2*
IFNβ	*Ifit1bl1,Ifi213, Ms4a4c, Phf11d, Mmp13, Phf11b, Kdr, Ifit2, Isg20, Herc6, Gadd45g, Mov10, Ddx60, Itpr1, Ifit3b, Oasl2, Abcg1, Tlr3, Ifi206, Slc28a2, Ifih1, Ifi44, Usp18, Ifit3, Mndal, Ifi209, Il1rn, Oasl1, Irf7, Nt5c3, Thbs1, Sp140, Rsad2, Slfn5, Ifi208, Cmpk2, Ifit1*
Macrophage Differentiation	*Adgre1, Csf1r, H2-Ab1, H2-Aa, Cd68, Lyz2, Itgam, Mertk, C1qa, C1qb, C1qc*
Monocyte*	*S100a4, Itgb7, Napsa, Cd300lg, Adora2b, Emb, Ly6c2, Ms4a4c, Fn1, Sell, Padi2, Lilra6, Ccnb2, Galnt9, Upb1, Lmo1, F13a1, Ccr2, Gm15987*
Notch Signaling	*Jag1, Notch2, Bmp2k, Dtx3l, Adam10, Adam17, Hdac1, Psen2*
Oxidative Phosphorylation	*Cox5a, Cox6c, Cox7c, Ndufa12, Ndufc1, Ndufs7, Uqrcb, Ndufs3, Atp5b, Atp6ap2, Prdx5, Txn1, Gsr, Ptgs2, Ccs, Prdx6, Gpx4, Sesn1, Sod3, Sod2, Ltc4s, Cox6b1*
Phagosome Formation	*Fcgr3, Fcgr1, Itga4, Itgb2, Coro1a, Kras, Rac2, Itgal, Rap1a, Rap1b*
Production of nitric oxide & reactive oxygen species	*Apoe, Ifngr1, Ikbkb, Nfkb1, Tnfrsf1a, Tnfrsf1b, Map3k14, Ncf2, Nos2, Nox2, Plcg2*

Table S3: VIZGEN PROBES

Gene	ensembl	transcript	target_regions	isoform_length
Adam19	ENSMUSG00000011256	11400	163	6383
Apoe	ENSMUSG00000002985	174355	33	1104
Atf2	ENSMUSG00000027104	173010	50	1658
Atf3	ENSMUSG00000026628	27941	59	1984
Atf4	ENSMUSG00000042406	109605	51	1725
C1qa	ENSMUSG00000036887	46285	30	1040
C1qb	ENSMUSG00000036905	46384	33	1088

C1qc	ENSMUSG00000036896	46332	34	1203
Car4	ENSMUSG00000000805	103194	38	1256
Ccl22	ENSMUSG00000031779	34231	67	2216
Ccl6	ENSMUSG00000018927	19071	41	1440
Ccl9	ENSMUSG00000019122	19266	66	3006
Ccr2	ENSMUSG00000049103	168841	62	5728
Ccr7	ENSMUSG00000037944	103134	60	1978
Cd14	ENSMUSG00000051439	61829	49	1641
Cd2	ENSMUSG00000027863	29456	35	1153
Cd38	ENSMUSG00000029084	30964	74	3270
Cd40	ENSMUSG00000017652	17799	81	2986
Cd63	ENSMUSG00000025351	220245	52	2894
Cd69	ENSMUSG00000030156	205032	109	4968
Cd74	ENSMUSG00000024610	50487	36	1224
Cd83	ENSMUSG00000015396	15540	66	2477
Cd86	ENSMUSG00000022901	89620	63	2539
Clec4a2	ENSMUSG00000030148	41779	54	2481
Clec4e	ENSMUSG00000030142	32239	69	2518
Clec7a	ENSMUSG00000079293	112076	62	2277
Col4a1	ENSMUSG00000031502	33898	152	6615
Coro1a	ENSMUSG00000030707	32949	46	1660
Coro1b	ENSMUSG00000024835	8893	53	1888
Crebbp	ENSMUSG00000022521	23165	69	10820
Csf3r	ENSMUSG00000028859	30673	65	3160
Cx3cr1	ENSMUSG00000052336	177637	93	3156
Cxcl2	ENSMUSG00000058427	200919	51	2066
Cxcr2	ENSMUSG00000026180	27372	66	2220
Cxcr4	ENSMUSG00000045382	52172	48	1805
Dnase1l3	ENSMUSG00000025279	26315	139	4747
Dpp4	ENSMUSG00000035000	47812	106	5268
Dusp1	ENSMUSG00000024190	25025	51	1990
Eif4e	ENSMUSG00000028156	29803	103	4991
F13a1	ENSMUSG00000039109	224446	57	2965
Fabp4	ENSMUSG00000062515	191757	102	4231
Fcgr1	ENSMUSG00000015947	29748	58	2589
Fcgr4	ENSMUSG00000059089	78825	37	1256
Fos	ENSMUSG00000021250	21674	58	2108
H2-Aa	ENSMUSG00000036594	173944	66	3223
H2-Ab1	ENSMUSG00000073421	40828	36	1212

H2-D1	ENSMUSG00000073411	172785	33	2102
H2-Dma	ENSMUSG00000037649	237402	61	2005
H2-Eb1	ENSMUSG00000060586	235530	44	1669
H2-K1	ENSMUSG00000061232	114311	31	1874
Hif1a	ENSMUSG00000021109	21530	86	4724
Hmox1	ENSMUSG00000005413	5548	30	1569
Hspa8	ENSMUSG00000015656	149936	31	3158
Icam2	ENSMUSG00000001029	1055	39	1282
Icosl	ENSMUSG00000000732	105393	85	2814
Ifnar1	ENSMUSG00000022967	23689	112	3909
Ifnar2	ENSMUSG00000022971	23693	59	3047
Ifng	ENSMUSG00000055170	68592	32	1208
Ifngr1	ENSMUSG00000020009	20188	62	2112
Ifngr2	ENSMUSG00000022965	23687	99	3708
Il1a	ENSMUSG00000027399	28882	56	1974
Il1b	ENSMUSG00000027398	28881	35	1356
Il1r2	ENSMUSG00000026073	27243	44	1419
Irak2	ENSMUSG00000060477	59286	63	3196
Irak3	ENSMUSG00000020227	20448	61	3097
Irf1	ENSMUSG00000018899	108920	65	2137
Irf4	ENSMUSG00000021356	21784	140	4764
Irf5	ENSMUSG00000029771	4392	59	2189
Irf7	ENSMUSG00000025498	26571	56	1916
Irf8	ENSMUSG00000041515	162001	66	2270
Irf9	ENSMUSG00000002325	145680	50	3869
Irs2	ENSMUSG00000038894	40514	170	6323
Itga6	ENSMUSG00000027111	126787	71	2721
Itgal	ENSMUSG00000030830	106306	93	5195
Itgax	ENSMUSG00000030789	33053	75	4046
Jak2	ENSMUSG00000024789	65796	136	5030
Jun	ENSMUSG00000052684	107094	85	3189
Junb	ENSMUSG00000052837	64922	42	1809
Krt79	ENSMUSG00000061397	230441	68	2451
Lamp1	ENSMUSG00000031447	33824	62	2229
Lars2	ENSMUSG00000035202	214557	58	2441
Lgals3	ENSMUSG00000050335	146468	31	1072
Ly6c2	ENSMUSG00000022584	187864	57	2680
Ly6e	ENSMUSG00000022587	51698	69	2281
Lyve1	ENSMUSG00000030787	33050	58	2893

Mafb	ENSMUSG00000074622	99126	80	3363
Marco	ENSMUSG00000026390	27639	56	1925
Mmp9	ENSMUSG00000017737	17881	85	3175
Mrc1	ENSMUSG00000026712	28045	129	5374
Ms4a7	ENSMUSG00000024672	162785	66	3607
Myl6	ENSMUSG00000090841	218813	80	3040
Nfkb2	ENSMUSG00000025225	73116	86	2979
Nfkb2	ENSMUSG00000025225	73116	86	2979
Nos2	ENSMUSG00000020826	214397	104	3456
Nr4a1	ENSMUSG00000023034	23779	70	2477
Nr4a3	ENSMUSG00000028341	30025	101	5720
Plac8	ENSMUSG00000029322	112910	34	1224
Plaur	ENSMUSG00000046223	206636	54	2009
Plet1	ENSMUSG00000032068	114474	31	1689
Pparg	ENSMUSG00000000440	171644	53	2125
Scarf2	ENSMUSG00000012017	12161	76	3308
Sell	ENSMUSG00000026581	192047	102	5703
Serf2	ENSMUSG00000074884	139253	71	3056
Siglec1	ENSMUSG00000027322	28794	195	6387
Sirpa	ENSMUSG00000037902	179001	99	3812
Slamf1	ENSMUSG00000015316	15460	50	2693
Socs1	ENSMUSG00000038037	38099	70	2785
Socs3	ENSMUSG00000053113	54002	65	2552
Sod2	ENSMUSG00000006818	7012	106	5861
Sparc	ENSMUSG00000018593	108858	63	2115
Spn	ENSMUSG00000051457	49931	73	3743
Spp1	ENSMUSG00000029304	31243	40	1410
Stat1	ENSMUSG00000026104	186857	100	4241
Stat2	ENSMUSG00000040033	85708	132	4394
Tgfb1	ENSMUSG00000002603	2678	54	2175
Tgfb2	ENSMUSG00000039239	195201	89	3485
Tgfbr1	ENSMUSG00000007613	7757	78	5756
Tnf	ENSMUSG00000024401	25263	48	1639
Tra2b	ENSMUSG00000022858	161286	55	3403
Traf6	ENSMUSG00000027164	4949	159	6169
Trem2	ENSMUSG00000023992	132340	109	4784
Xcr1	ENSMUSG00000060509	182350	121	4453

Table S4. Antibodies for flow cytometry

Antibody	Clone	Source
CD11c-BV605	N418	BioLegend 117334
CD11b-BV711	M1/70	BioLegend 101241
Ly6G-BV421	1A8	BioLegend 127628
Ly6C-PE-Cy7	HK1.4	BioLegend 128018
I-A/I-E(MHCII)-PE-Dazzle	M5/114.15.2	BioLegend 107647
SiglecF-APC-Cy7	E50-2440	BD Biosciences 565527
CD90.2-PE-Cy5	30-H12	BioLegend 105314
CD19-PE-Cy5	6D5	BioLegend 115510
NK1.1-PE-Cy5	PK136	BioLegend 108716
CD45-PE	104	BioLegend 109808
CD45.2-PerCp/Cy5.5	104	Biolegend 109827

Data S1. (separate file)

Mean fluorescence intensity (MFI) measurements of cells sorted for single-cell RNA sequencing. The MFI values for cell size and antibodies used during sorting (refer to FigS1) are provided for each cell. Cell type identification was performed using Boolean expressions of gating parameters applied during sorting with the Sony MA900 software.

Data S2. (separate file)

Differential expressed analysis of genes between Leiden subclusters within SmartSeq2 (SS2) atlas. Differentially expressed genes between Leiden subclusters of SS2 atlas were determined using MAST in R. Data is organized in separate Excel tabs for each subcluster as indicated in Figure 2D. Subcluster 0 serves as the reference subcluster as it is the largest quantity of cells.

Data S3. (separate file)

Pathway analysis of SS2 Leiden subclusters. Using differentially expressed genes determined from MAST with an FDR <0.1, gene ontology and pathway analysis were conducted using multiple reference libraries including ShinGOv0.61, EnrichR, and KEGG (R). These results highlight key differences of gene ontology between clusters. The data from Data S2 can be utilized to replicate the pathway analysis with other packages of interest.

Data S4. (separate file)

Differential expressed analysis of transitioning monocytes between different infection conditions of the SS2 atlas. MAST (R) facilitated the identification of differentially expressed genes among transitioning monocytes isolated from uninfected mice, mice infected with H37Rv, and mice infected with H37RvΔEccD1. Data is organized with each comparison separated by Excel tabs.

Data S5. (separate file)

Differential gene expression of monocyte-derived macrophages in mice infected with H37RvΔEccD1 compared to those infected with H37Rv as analyzed by MAST. Differentially expressed genes with an FDR <0.1 were identified and used for pathway analysis and gene ontology. Results are segregated by tabs using different computational packages.

Data S6. (separate file)

Differential gene expression analysis of bone marrow-derived macrophages (BMDM). BMDM were infected with H37Rv or H37RvΔRD1 for 24 hours. The comparison of single-cell gene expression was done using MAST (R), with each comparison detailed in separate Excel tabs.
